# Broad Distribution of TPI-GAPDH Fusion Proteins among Eukaryotes: Evidence for Glycolytic Reactions in the Mitochondrion?

**DOI:** 10.1371/journal.pone.0052340

**Published:** 2012-12-20

**Authors:** Takuro Nakayama, Ken-ichiro Ishida, John M. Archibald

**Affiliations:** 1 Department of Biochemistry & Molecular Biology, Canadian Institute for Advanced Research, Program in Integrated Microbial Biodiversity, Dalhousie University, Halifax, Nova Scotia, Canada; 2 Faculty of Life and Environmental Sciences, University of Tsukuba, Tsukuba, Ibaraki, Japan; University of Melbourne, Australia

## Abstract

Glycolysis is a central metabolic pathway in eukaryotic and prokaryotic cells. In eukaryotes, the textbook view is that glycolysis occurs in the cytosol. However, fusion proteins comprised of two glycolytic enzymes, triosephosphate isomerase (TPI) and glyceraldehyde-3-phosphate dehydrogenase (GAPDH), were found in members of the stramenopiles (diatoms and oomycetes) and shown to possess amino-terminal mitochondrial targeting signals. Here we show that mitochondrial TPI-GAPDH fusion protein genes are widely spread across the known diversity of stramenopiles, including non-photosynthetic species (*Bicosoeca* sp. and *Blastocystis hominis*). We also show that TPI-GAPDH fusion genes exist in three cercozoan taxa (*Paulinella chromatophora*, *Thaumatomastix* sp. and *Mataza hastifera*) and an apusozoan protist, *Thecamonas trahens*. Interestingly, subcellular localization predictions for other glycolytic enzymes in stramenopiles and a cercozoan show that a significant fraction of the glycolytic enzymes in these species have mitochondrial-targeted isoforms. These results suggest that part of the glycolytic pathway occurs inside mitochondria in these organisms, broadening our knowledge of the diversity of mitochondrial metabolism of protists.

## Introduction

Glycolysis is a fundamental catabolic biochemical pathway occurring in essentially all living cells. The pathway produces two pyruvate molecules from one glucose molecule and yields two ATP. In eukaryotic cells, the sequential reactions of the glycolytic pathway generally occur in the cytosol and the end product, pyruvate, is transported to the mitochondrion for subsequent participation in the TCA cycle. This textbook version of eukaryotic glycolysis is based mainly on studies of plants and animals, although evidence suggests that at least some unicellular eukaryotes–protists–deviate from the canonical glycolytic pathway. For example, atypical compartmentalization of glycolytic enzymes is seen in trypanosomatid protists (i.e., *Trypanosoma* spp. and *Leishmania* spp.), well known as the causative agents of human diseases [1], [2]. Trypanosomatids possess a peroxisome-like structure called a glycosome and enzymes involved in the first part of glycolysis (from hexokinase to phosphoglycerate kinase) function in this membrane-enclosed compartment [3], [4], [5], [6].

A glycolytic enzyme showing a non-canonical localization has also been found in diatom algae. In addition to cytosolic triosephosphate isomerase (TPI) and glyceraldehyde-3-phosphate dehydrogenase (GAPDH) enzymes, diatoms possess an isoform of both proteins in the form of a fusion protein, TPI-GAPDH [7]. The fusion protein contains an intact TPI moiety at its amino- (N-) terminus followed by an entire GAPDH sequence. Interestingly, the fusion protein has a N-terminal extension resembling a mitochondrial targeting signal. The TPI-GAPDH fusion has been shown to localize to the mitochondrial matrix by immuno-electron microscopy in the diatom *Phaeodactylum tricornutum* [7].

GAPDH is a ubiquitous enzyme with important roles mainly in glycolysis and photosynthesis. In glycolysis, GAPDH catalyzes the reversible conversion of glyceraldehyde-3-phosphate and glycerate-1,3-bisphosphate. TPI carries out a glycolytic reaction just before that of GAPDH, i.e., conversion of dihydroxyacetone phosphate to glyceraldehyde-3-phosphate. Thus, TPI and GAPDH play a role in sequential reactions. Proteins involved in glycolysis are often encoded in operons in prokaryotes [8], [9], and a transcriptional fusion of two glycolytic enzyme genes (GAPDH and enolase) exists in dinoflagellate algae [10]. In addition to the TPI-GAPDH fusion protein, Liaud et al. (2000) [7] showed that one of two phosphoglycerate kinase (PGK) isoforms in *P. tricornutum* has a N-terminal mitochondrial targeting signal. These authors concluded that glycolytic enzymes functioning ‘downstream’ of TPI and GAPDH exist in diatom mitochondria, i.e., pyruvate produced in the mitochondrion is fed directly into the TCA cycle. This interpretation is supported by recent genomic analysis of *P. tricornutum* [11].

A TPI-GAPDH fusion protein gene has also been found in oomycetes, plastid-lacking protists best known as plant pathogens [12]. Diatoms and oomycetes belong to the same lineage of eukaryotes, the stramenopiles. The stramenopiles are a phylogenetically diverse group of eukaryotes and include a large photosynthetic lineage called heterokontophytes, organisms that acquired their plastid through secondary endosymbiosis with a red alga [13], [14]. Diatoms, brown algae, chrysophytes and several plastid-bearing groups all belong to the heterokontophytes to which oomycetes are specifically but only distantly related [15]. Interestingly, a TPI-GAPDH fusion gene was recently found in the genome of another heterokontophyte, the brown alga *Ectocarpus siliculosus* [16], [17]. Here we show that TPI-GAPDH fusion genes are widely spread across the known diversity of stramenopiles. We also present evidence for the existence of TPI-GAPDH fusion genes in three cercozoan species and an apusozoan protist; none of these organisms belong to the stramenopiles. Subcellular localization predictions for glycolytic enzymes in these TPI-GAPDH fusion-containing protists support the hypothesis that at least part of the glycolytic pathway occurs inside their mitochondria, a curious finding in light of conventional views on the evolution of mitochondrial metabolism.

**Figure 1:**
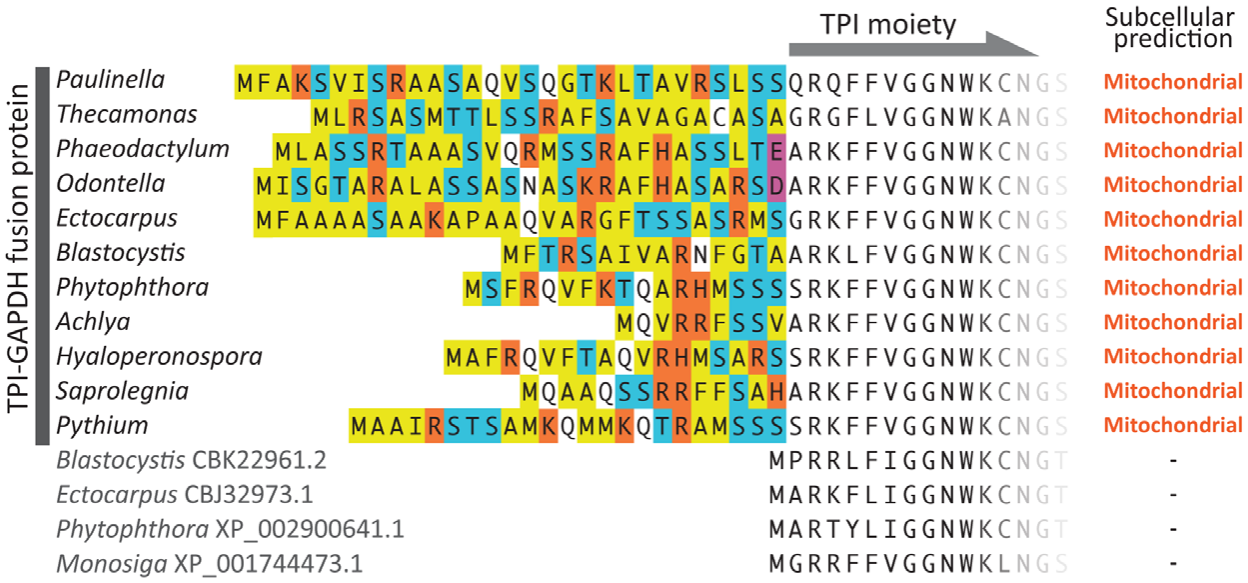
N-terminal leader sequences of TPI-GAPDH fusion proteins. Alignment of the N-terminal region of TPI-GAPDH fusion proteins with four stand-alone TPI proteins. Amino acid residues on the leader sequences are colored as follows. Yellow: hydrophobic residues, blue: hydroxylated residues, orange: positively charged residues, purple: negatively charged residues and white: other polar uncharged residues. Results of prediction analysis are summarized on the right side of sequences. “Mitochondrial” suggests a mitochondrial localization based on the results of four different prediction programs (see main text).

## Materials and Methods

### Detection of TPI-GAPDH Fusion Protein Gene Sequences

Total DNA of *Paulinella chromatophora* strain M0880/a, which was kindly provided by Dr. Michael Melkonian (University of Cologne, Germany), was extracted using the DNeasy Plant Mini Kit (QIAGEN) and a TPI-GAPDH fusion protein gene sequence was amplified by PCR with primers 5′-GAYGCNCARTAYATGGCNTAYATG-3′ and 5′-TANCCCCAYTCGTTRTCRTACCA-3′. To determine the 5′ end of the gene, we performed rapid amplification of cDNA ends (RACE) on total RNA of strain M0880/a that had been extracted using the RNeasy Plant Mini Kit (QIAGEN). RACE reactions were carried out using a 5′ RACE System (Invitrogen) according to manufacturer’s instructions and with exact-match primers. Introns in the *P. chromatophora* TPI-GAPDH protein gene sequence were detected by aligning genomic and cDNA sequences, and by comparing the deduced protein sequence with TPI-GAPDH protein sequences from other organisms.

**Figure 2:**
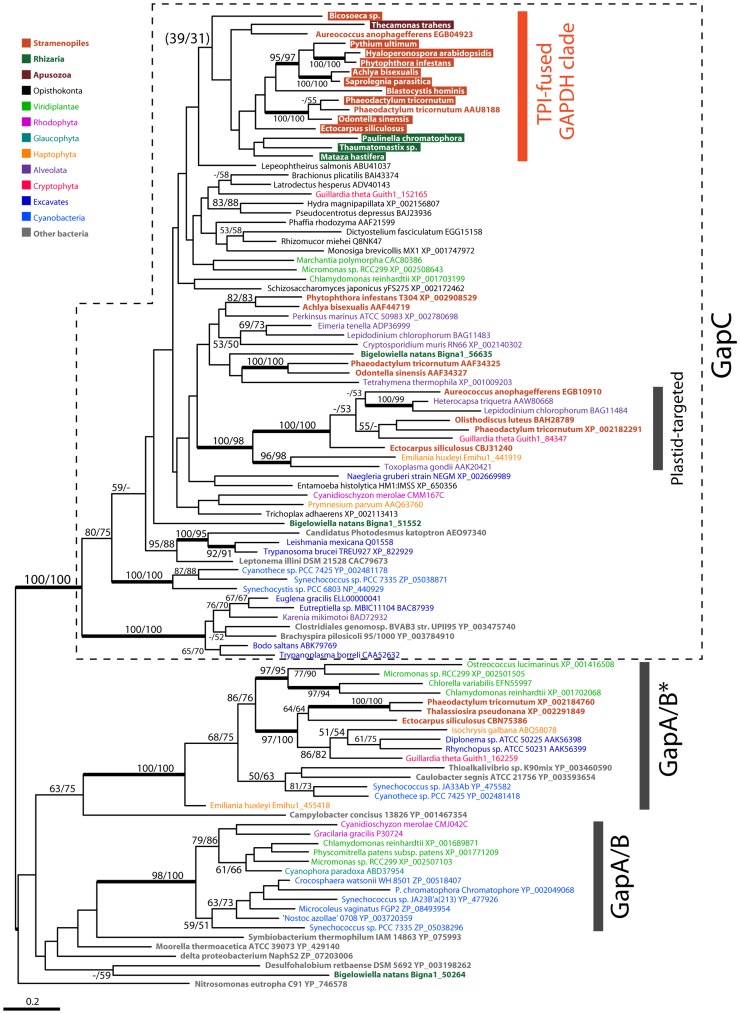
Maximum likelihood tree of GAPDH protein sequences. GAPDH ML tree constructed using RAxML with the LG+I+G+F model. GAPDH sequences fused with TPI are highlighted by colored boxes. The numbers at nodes are ML bootstrap values (left: RAxML, right: PhyML). Bootstrap values below 50% are not shown except values for nodes of particular interest. Nodes received bootstrap values ≥90% in both RAxML and PhyML analyses are indicated by thick line. The root was arbitrarily chosen for display purposes. Scale bar shows the number of inferred amino acid substitutions per site.

For *Bicosoeca* sp. NIES-1438, *Thaumatomastix* sp. NIES-2378 and *Mataza hastifera* NIES-2568, cDNAs were kindly provided by Dr. Ryoma Kamikawa and Dr. Yuji Inagaki [18]. Partial TPI-GAPDH sequences were amplified from cDNA by PCR using the degenerate primers 5′-GGNGGNAACTGGAARTGYAAYGG-3′ and 5′-TANCCCCAYTCRTTRTCRTACCA-3′. New sequences obtained in this study are available in GenBank (accession numbers: JQ783118-JQ783122). TPI-GAPDH protein gene sequences in the publicly available genomes of *Thecamonas trahens*, *Pythium ultimum* DAOM BR144, *Saprolegnia parasitica* CBS 223.65 and *Hyaloperonospora arabidopsidis* Emoy2 were detected with tblastn searches using the TPI-GAPDH fusion of *Phaeodactylum tricornutum* (GenBank accession: AAF34330) as a query. GenBank accession numbers for each contig containing the TPI-GAPDH genes are as follows: ADVD01000324, ADOS01000576, ADCG01002396 and ABWE01000065, respectively. Sequences from the genome of the diatom *Thalassiosira pseudonana* [19] were not used in this study due to uncertainties with the gene models in their N-terminal coding regions.

**Table 1:** Numbers of putative mitochondrial-targeted glycolytic enzyme isoforms in stramenopiles and the chlorarachniophyte *Bigelowiella natans*.

Organism	PGK	PGAM	Enolase	PK
*Phaeodactylum tricornutum*	**1** (3)	**1** (6)	**2** (3)	**2** (8)
*Aureococcus anophagefferens*	**1** (2)	**1** (11)	0 (3)	**2** (6)
*Ectocarpus siliculosus*	**1** (5)	**1** (6)	**1** (2)	0 (3)
*Phytophthora infestans*	**1** (1)	**1** (2)	**1** (3)	**1** (5)
*Blastocystis hominis*	**1** (1)	0 (1)	**1** (2)	0 (1)
*Bigelowiella natans*	0 (2)	**2** (10)	0 (2)	**2** (8)

Putative mitochondrion-targeted glycolytic enzymes were inferred from localization predictions using TargetP, iPSORT, Predotar and PredSL. Numbers in parentheses show the total numbers of isoforms tested in this study.

### Mitochondrial Targeting Prediction of Glycolytic Enzymes

Phosphoglycerate kinase (PGK), phosphoglycerate mutase (PGAM), enolase and pyruvate kinase (PK) sequences from 5 stramenopiles (*Phaeodactylum tricornutum* CCAP 1055/1, *Aureococcus anophagefferens*, *Ectocarpus siliculosus*, *Phytophthora infestans* T30-4 and *Blastocystis hominis*) were collected using blastp searches against public protein databases with a threshold of 1e**–**50. Sequences from *Phaeodactylum tricornutum* CCAP 1055/1 were used as queries (GenBank accession numbers XP_002183701, XP_002185492, XP_002176181 and XP_002183584 for PGK, PGAM, enolase and PK, respectively). Sequences for these four proteins were also retrieved from the genome of the chlorarachniophyte alga *Bigelowiella natans* CCMP2275 via the Joint Genome Institute (JGI) database (http://genome.jgi-psf.org/Bigna1/Bigna1.home.html). *B. natans* proteins corresponding to PGK, PGAM, enolase and PK (KOG IDs: KOG1367, KOG0235, KOG2670 and KOG2323, respectively) were retrieved, several of which were removed from the dataset due to concerns over their orthology.

**Figure 3:**
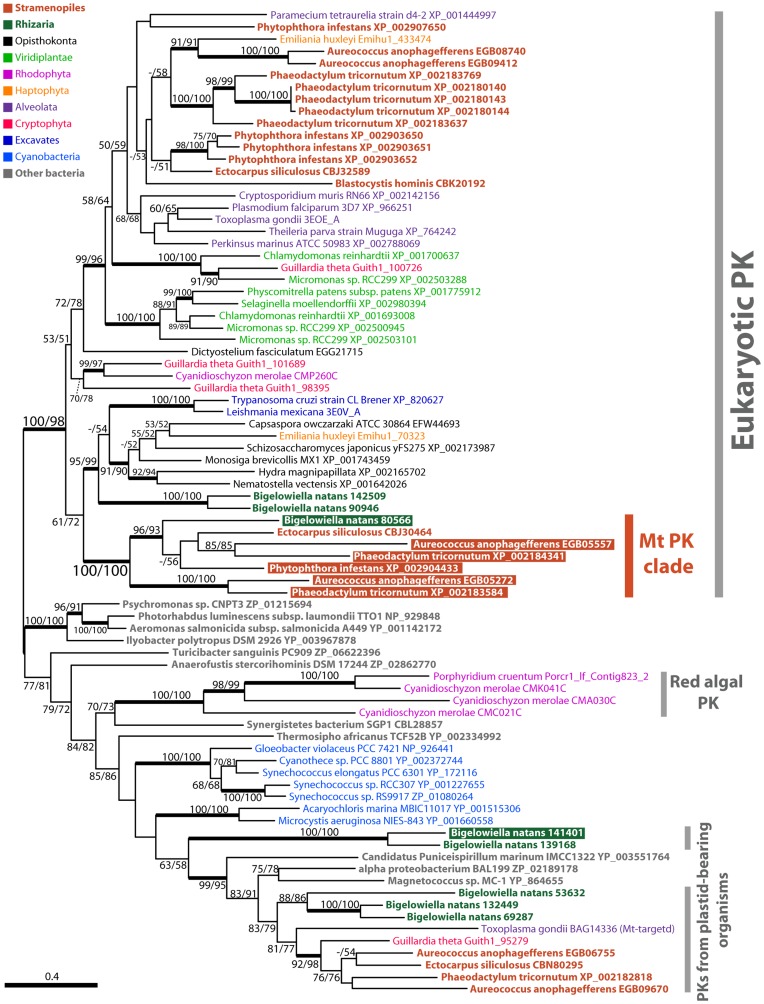
Maximum likelihood tree of PK protein sequences. PK tree constructed using RAxML with the LG+G model. Putative mitochondrial-targeted PK sequences are highlighted by colored boxes. All other details are as in Figure 2.

The N-termini of stramenopile and *B. natans* protein sequences were analyzed using TargetP [20], iPSORT [21], Predotar [22] and PredSL [23], using both the ‘plant’ and ‘non-plant’ settings for each program. Only proteins predicted to be targeted to mitochondria with all four programs and with both settings were considered strong candidates for being organelle localized in their respective organisms.

**Figure 4:**
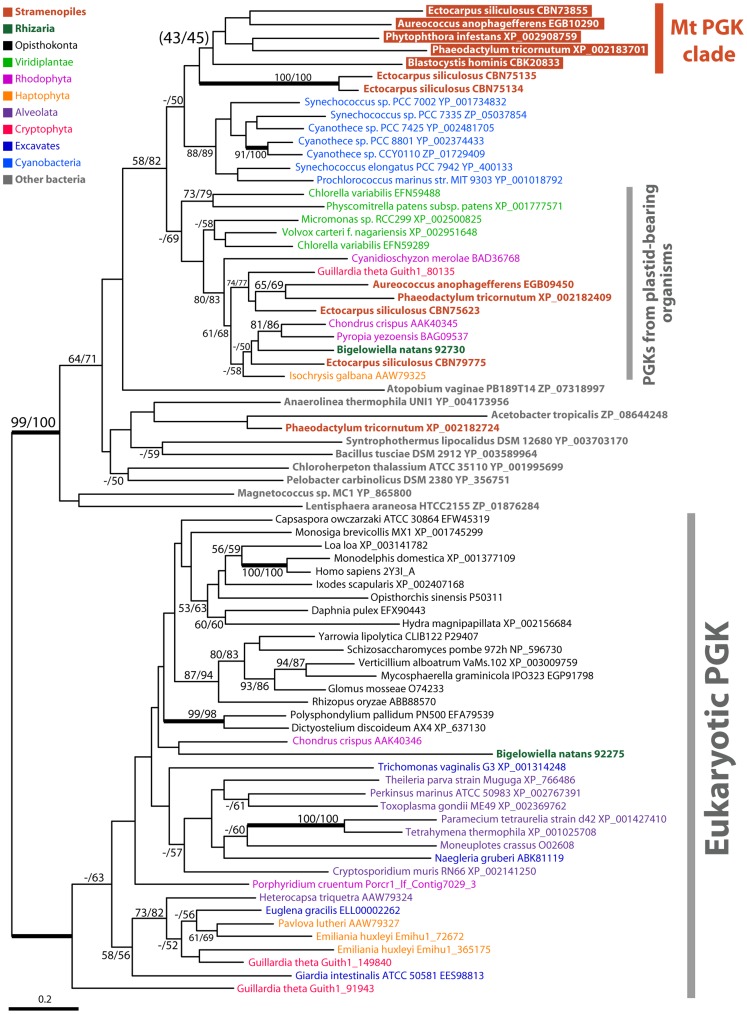
Maximum likelihood tree of PGK protein sequences. PGK tree constructed using RAxML and the LG+I+G+F model. Putative mitochondrial-targeted PGK sequences are highlighted by colored boxes. See Figure 2 for other presentation details.

### Phylogenetic Analyses

TPI-GAPDH protein sequences deduced from DNA sequences in this study were aligned individually with TPI and GAPDH sequences from a broad diversity of eukaryotes and bacteria. Stramenopile and *B. natans* glycolytic enzyme sequences were also aligned with their orthologs from eukaryotes and prokaryotes. Highly diverged sequences and ambiguously aligned positions were manually removed from alignments prior to phylogenetic analyses. A complete list of the sequences used in our analyses is provided in Table S1. Each alignment was analyzed using ModelGenerator [24], based on Akaike Information Criterion 1 (AIC1), to estimate the most appropriate model of protein sequence evolution for each phylogenetic analysis. Maximum likelihood (ML) phylogenetic analyses were performed using RAxML 7.2.6 [25] with evolutionary models chosen by ModelGenerator. Searches for the best trees were conducted starting from 10 random trees. Bootstrap support was evaluated with non-parametric bootstrapping using 100 replicates with both RAxML and PhyML [26]. Both bootstrap analyses were run with the same model used in each ML analysis.

**Table 2:** Summary of putative mitochondrial-targeted glycolytic enzymes in diverse eukaryotes.

Supergroup	Subgroup	Species	Mt-targeted TPI-GAPDH fusion protein	Mt-targeted PGK	Mt-targeted PGAM	Mt-targeted enolase	Mt-targeted PK
Stramenopiles	Heterokontophytes	*Phaeodactylum tricornutum* p	**Detected**	**Detected**	**Detected**	**Detected**	**Detected**
		*Aureococcus anophagefferens* p	Not detected	**Detected**	**Detected**	Not detected	**Detected**
		*Ectocarpus siliculosus* p	**Detected**	**Detected**	**Detected**	**Detected**	Not detected
	Oomycetes	*Phytophthora infestans*	**Detected**	**Detected**	**Detected**	**Detected**	**Detected**
		*Hyaloperonospora arabidopsidis*	**Detected**	–	–	–	–
		*Saprolegnia parasitica*	**Detected**	–	–	–	–
		*Pythium ultimum*	**Detected**	–	–	–	–
	Bicosoecea	*Bicosoeca* sp.	**Detected** *	–	–	–	–
	Blastocystae	*Blastocystis hominis*	**Detected**	**Detected**	Not detected	**Detected**	Not detected
Rhizaria	Cercozoa	*Bigelowiella natans* p	Not detected	Not detected	**Detected**	Not detected	**Detected**
		*Paulinella chromatophora* p	**Detected**	–	–	–	–
		*Thaumatomastix* sp.	**Detected** *	–	–	–	–
		*Mataza hastifera*	**Detected** *	–	–	–	–
Apusozoa	Apusomonadidae	*Thecamonas trahens*	**Detected**	–	–	–	–

* Targeting of the proteins is unknown because the N-terminal sequences were not available.

p Photosynthetic organisms.

- Not examined.

## Results

### TPI-GAPDH Fusion Protein Genes from Stramenopiles, Cercozoans and an Apusozoan

As seen in the diatom *Phaeodactylum tricornutum* [7], we found three TPI-GAPDH fusion protein gene sequences in the genome of the stramenopile parasite, *Blastocystis hominis*, which has a mitochondrion-like organelle (MLO) [27]. Although the three *B. hominis* sequences (GenBank accession numbers CBK20353, CBK22421 and CBK23790) were annotated as stand-alone GAPDH sequences, comparison to other TPI-GAPDH proteins clearly revealed the existence of complete TPI moieties upstream of all three GAPDH coding regions. The three TPI-GAPDH protein sequences from *B. hominis* were 99% identical to each other. In addition, we obtained a TPI-GAPDH protein gene sequence from mRNA of *Bicosoeca* sp., a free-living heterotrophic stramenopile, and also detected the gene in the whole genome shotgun (WGS) sequences of three oomycetes, *Hyaloperonospora arabidopsidis* Emoy2, *Pythium ultimum* DAOM BR144 and *Saprolegnia parasitica* CBS 223.65. In contrast, we could not find a TPI-GAPDH gene sequence in the complete genome sequence of the heterokontophyte *Aureococcus anophagefferens* [28].

Interestingly, we also identified TPI-GAPDH protein genes in three cercozoans, *Paulinella chromatophora*, *Thaumatomastix* sp. and *Mataza hastifera* [29] using RT-PCR. However, examination of the recently sequenced nuclear genome of the photosynthetic cercozoan, *Bigelowiella natans* CCMP2275 (http://genome.jgi-psf.org/Bigna1/Bigna1.home.html) did not uncover a TPI-GAPDH gene. Only stand-alone versions of these enzymes were identified. Finally, a search of the genome sequence of the apusozoan *Thecamonas trahens* uncovered a TPI-GAPDH protein gene.

### Mitochondrial Targeting Signals in TPI-GAPDH Fusion Proteins

Figure 1 shows an alignment of the N-terminal region of TPI-GAPDH proteins. With the exception of *Ectocarpus siliculosus*, all TPI-GAPDH proteins derived from complete gene sequences possessed obvious N-terminal extensions (the *Bicosoeca* sp., *Thaumatomastix* sp. and *Mataza hastifera* sequences were not included because the 5′ ends of their respective cDNA were not obtained). These leader sequences were rich in hydrophobic amino acid residues such as alanine and valine, as well as the hydroxylated residues serine and threonine. Positively charged amino acid residues were well represented while negatively charged amino acids were rare. These characteristics are consistent with the properties of mitochondrial targeting signals [30], [31]. In addition, RXXS motifs, which are frequently observed at cleavage sites of the mitochondrial targeting signal peptides (R-2 group [31]), were found in the latter portions of the N-terminal extensions of *P. chromatophora* and all the oomycetes sequences. Positive mitochondrial-targeting predictions were obtained using four different subcellular localization prediction programs (TargetP, iPSORT, Predotar and PredSL) with the ‘non-plant’ setting (Figure 1, for detailed results see Table S2). By close investigation of the gene model for the fusion protein sequence of *Ectocarpus siliculosus* (locus tag: Esi_0187_0027), we identified a potential translation initiation codon 78 bp upstream of the originally predicted open reading frame. When this alternative start codon was selected, the N-terminal sequence of the protein was extended 26 amino acid residues. Interestingly, this extended N-terminal sequence showed a similar amino acid composition to the other TPI-GAPDH sequences from other stramenopiles and rhizarians (Figure 1). Further, this leader sequence was also predicted to be a mitochondrial targeting signal by four subcellular localization prediction programs (Figure 1, Table S2).

### TPI-GAPDH Fusion Protein Phylogeny

In an attempt to infer the evolution of the TPI-GAPDH fusion proteins in the diverse collection of eukaryotes that bear them, we constructed phylogenetic trees separately for the TPI and GAPDH moieties. Several distinct GAPDH isoforms exist in eukaryotes, more than one of which can exist within the same cell (e.g., phototrophs possess both cytosolic and plastid-targeted GAPDH proteins). Therefore, we examined the full spectrum of GAPDH sequence diversity in our analysis of the TPI-GAPDH fusion proteins. In the GAPDH tree (Figure 2), all TPI-fused GAPDH sequences branched within the “GapC” clade, and formed a monophyletic group within it, albeit without bootstrap support. Investigation of bootstrap partitions revealed that the positions of the *Thecamonas trahens* and *Bicosoeca* sp. sequences were unstable. We therefore repeated the analysis with these two sequences excluded. In this modified analysis bootstrap support for the monophyly of the TPI-fused GAPDH sequences in ML analysis was 92% with RAxML and 86% with PhyML (Figure S1). This clade also contained two GAPDH sequences that are not fused with TPI, one from *Aureococcus anophagefferens* (EGB04923) and another from *Phaeodactylum tricornutum* (AAU81889). While the *P. tricornutum* GAPDH was shown to have a mitochondrial targeting signal despite not being fused with TPI [11], we could detect no obvious N-terminal extension on the *A. anophagefferens* sequence.

As in previous studies, our phylogenies show that the plastid-targeted GAPDH proteins of stramenopiles form a robust clade among GapC sequences together with those of “chromalveolate” organisms (Figures 2 and S1). The phylogenetic position of the stramenopile cytosolic GAPDH sequences is not well resolved in our analyses but nevertheless branched in the GapC clade with cytosolic GAPDH from other eukaryotes.

Rogers et al. [32] showed that some diatom GAPDH sequences branch within a clade of poorly characterized GAPDH isoforms known as “GapA/B*”. The GapA/B* clade was shown to contain only sequences from diatoms, a haptophyte and diplonemids. However, with improved taxon sampling, the GapA/B* clade in our tree now also contains GAPDH sequences from *Ectocarpus siliculosus*, the cryptophyte *Guillardia theta*, prasinophytes and chlorophytes. This clade received strong support and sequences from chromalveolates and diplonemids formed a robust monophyletic clade.

In TPI phylogenies, eukaryotic TPI sequences formed two distinct clades (eukaryotic TPI clades I and II, Figure S2). All GAPDH-fused sequences formed a monophyletic group within the eukaryotic TPI clade I (Figure S2), although the GAPDH-fused TPI clade lacked bootstrap support. Removal of the *Bicosoeca* sp. and *Thecamonas trahens* sequences had no effect on tree topology or support. The TPI-GAPDH clade also contained a TPI sequence from the alveolate *Perkinsus marinus* that is not fused with GAPDH. An obvious mitochondrial targeting signal was not found on the N-terminus of the *P*. *marinus* sequence.

### Mitochondrial Targeting Signals in Other Glycolytic Enzymes

Liaud et al. (2000) [7] showed that in the diatom *Phaeodactylum tricornutum*, phosphoglycerate kinase (PGK), an enzyme that functions immediately downstream of GAPDH in glycolysis, also possesses a mitochondrial targeting signal. A more recent genome-wide investigation of this organism showed that some isoforms of cofactor-dependent phosphoglycerate mutase (dPGAM), enolase and pyruvate kinase (PK) also possess mitochondrial targeting signals [11], suggesting that the latter part of glycolysis occurs inside of the *Phaeodactylum tricornutum* mitochondrion. The broad distribution of mitochondrial-targeted TPI-GAPDH protein genes raises the possibility that other TPI-GAPDH containing organisms also possess mitochondrial glycolytic enzymes. Therefore we retrieved sequences for four glycolytic enzymes functioning downstream of GAPDH (i.e., PGK, dPGAM, enolase and PK) from complete or partial genome data of four stramenopiles (*Ectocarpus siliculosus*, *Aureococcus anophagefferens*, *Phytophthora infestans* T30-4 and *Blastocystis hominis*) in addition to *Phaeodactylum tricornutum* CCAP 1055/1. We then determined whether or not these proteins have predicted mitochondrial targeting signals on their N-termini. For cercozoan species, we retrieved the same set of enzymes from *Bigelowiella natans* but not *Paulinella chromatophora*, as we could not find intact sequences for any of these proteins from transcriptome data [33], [34]. Some organisms are known to have an alternative form of PGAM, a cofactor-independent phosphoglycerate mutase (iPGAM) [35], [36], [37], however iPGAM sequences were not found in any of the above species.

In total we obtained and analyzed 96 protein sequences (Table S3). Subcellular localization predictions were conducted with four different prediction programs (TargetP, iPSORT, Predotar and PredSL); proteins were considered to be putative mitochondrial- or MLO-targeted proteins only if a positive result was obtained with all four programs with both the ‘plant’ and ‘non-plant’ settings. Combining results obtained with multiple prediction programs has been shown to reduce false-positives, albeit with an increase in false-negatives [38], [39].

In *P. tricornutum*, all four enzymes (PGK, PGAM, enolase and PK) are predicted to be mitochondrion-targeted (Table 1 and Table S3), consistent with the results of Kroth et al. (2008) [11]. In addition, *Phytophthora infestans* was predicted to have at least one mitochondrial-localized isoform for each of the four enzymes. As well, three enzymes from *Ectocarpus siliculosus* (PGK, PGAM and enolase) and *Aureococcus anophagefferens* (PGK, PGAM and PK), and two from *Blastocystis hominis* (PGK and enolase), were predicted to have mitochondrial- (or MLO-) targeted isoforms. Interestingly, the plastid-containing cercozoan *Bigelowiella natans* was also predicted to have a mitochondrial PGAM and PK, even though it does not possess the TPI-GAPDH fusion protein (Table 1 and Table S3).

### Phylogenies of Putative Mitochondrial Glycolytic Enzymes

To further investigate the evolution of mitochondrial targeted glycolytic enzymes, we constructed a phylogenetic tree for each protein. For PK, six of seven mitochondrial PK isoforms formed a robustly supported monophyletic clade (Figure 3). This PK clade includes enzymes from *Phaeodactylum tricornutum*, *Aureococcus anophagefferens, Phytophthora infestans* and *Bigelowiella natans*, and also contained a protein from *Ectocarpus siliculosus* that was not predicted to be localized to the mitochondrion in our analysis.

Similarly, although bootstrap support was weak, the PGK tree (Figure 4) showed a monophyletic clade of putative mitochondrial-targeted PGK enzymes from all five stramenopiles examined, including *Blastocystis hominis*. In contrast to the PK phylogeny, however, the mitochondrial PGK clade branched away from the major eukaryotic clade, instead branching together with cyanobacterial homologs and sequences from photosynthetic eukaryotes that are presumably derived from the plastid progenitor. The PGAM tree also contained a clade dominated by mitochondrion-localized enzymes (Figure S3) from four stramenopiles and two photosynthetic cercozoans, *Bigelowiella natans* and *Gymnochlora stellata*. Although the *G. stellata* PGAM was not detected as being a mitochondrial-targeted protein (data not shown), we cannot rule out this possibility based on the mRNA sequence data currently in hand. The phylogenetic position of the ‘mitochondrial’ clade of PGAM sequences in the context of the global phylogeny was not resolved.

In the enolase tree, all five of the mitochondrial-targeted enolase sequences we detected in *Phaeodactylum tricornutum*, *Ectocarpus siliculosus*, *Phytophthora infestans* and *Blastocystis hominis* branched robustly within the eukaryotic enolase clade (Figure S4), although they did not form a monophyletic group. Two mitochondrial isoforms from *P. tricornutum* branched with non-mitochondrial isoforms from stramenopiles as well as several enolase sequences from a haptophyte and dinoflagellate algae. Other mitochondrial enolase isoforms in stramenopiles branched at the base of this clade with weak support.

## Discussion

We have shown that TPI-GAPDH fusion proteins exist in *Bicosoeca* sp. and *Blastocystis hominis*, two ‘basal’ stramenopiles [15], as well as in heterokontophytes and oomycetes, implying a broad distribution within stramenopiles. Unexpectedly, the TPI-GAPDH fusion protein genes were also found in cercozoan species belonging to the ‘supergroup’ Rhizaria, as well as the apusozoan *Thecamonas trahens* whose exact phylogenetic position among eukaryotes is not known [40]. Phylogenetic analyses of the TPI and GAPDH moieties revealed that the TPI-GAPDH sequences cluster together. In the GAPDH tree, this clade is strongly supported when the *Thecamonas trahens* and *Bicosoeca* sp. sequences are removed (Figure S1). These results suggest that TPI-GAPDH fusion proteins, at least those from stramenopiles and cercozoans, share a common origin. This is also likely the case for the *T. trahens* and *Bicosoeca* sp. sequences if one believes that it is unlikely that the fusion event of the TPI and GAPDH genes and acquisition of the mitochondrial targeting signal, which is also found in almost all of the other TPI-GAPDH proteins, occurred independently in Apusozoa and Bicosoecida. On the other hand, we could not detect TPI-GAPDH proteins in draft genome sequences of the heterokontophyte alga *Aureococcus anophagefferens* and the photosynthetic cercozoan *Bigelowiella natans*. Nevertheless, despite the apparent absence of the fusion protein, one of two stand-alone GAPDH sequences in *Aureococcus anophagefferens* branches within the TPI-GAPDH clade and forms a monophyletic group with the fusion proteins of other heterokontophytes (Figure S1). Considering the fact that other heterokontophytes and stramenopiles possess the protein, it seems more likely that *A. anophagefferens* has secondarily lost the TPI-GAPDH fusion. In spite of its apparent origin, this particular *A. anophagefferens* GAPDH isoform does not have an obvious N-terminal extension compared to other GAPDH sequences (data not shown) and we could not find any other candidate sequence for another cytosolic form (i.e., an isoform lacking an N-terminal leader sequence) in the *A. anophagefferens* genome. Together, these observations suggest that GAPDH activity in the cytosol of *A. anophagefferens* has been taken over by a GAPDH that was once targeted to the mitochondrion.

The broad distribution of mitochondrial-targeted TPI-GAPDH proteins among stramenopiles is consistent with the notion that the fusion was present in the common ancestor of modern-day species. In addition, the existence of this protein in some rhizarians raises the intriguing possibility that the origin of the TPI-GAPDH fusion goes further back in time to a common ancestor shared by stramenopiles and rhizarians, two lineages that have been shown to have a robust affiliation (together with Alveolates) in recent multi-gene phylogenetic studies [41], [42], [43], [44], [45]. Nonetheless, whether early rhizarians truly possessed a TPI-GAPDH fusion is presently unclear because all three cercozoan species shown in this study to contain the fusion are members of a single subgroup of cercozoa (Monadofilosa [46]), and the fusion was not found in the draft genome of *Bigelowiella natans*, an organism that belongs to another cercozoan subgroup (Reticulofilosa [46]). It is difficult to trace the evolution of the TPI-GAPDH fusion between these two eukaryotic groups since neither the TPI nor GAPDH tree provides sufficient resolution on this point (Figure 2, S1 and S2). The presence of a TPI-GAPDH fusion in the apusozoan *Thecamonas trahens* is similarly intriguing and difficult to explain. The phylogenetic position of apusozoans on the tree of eukaryotes is controversial [40], [47], [48], [49]. Discrete characters such as gene fusions have the potential to help solve difficult phylogenetic problems [47], [50], [51], but are also notoriously difficult to interpret when data are sparse. The extent to which eukaryote-to-eukaryote horizontal gene transfer(s) should be evoked to explain the distribution of the TPI-GAPDH fusion proteins examined here remains an open question. The possibility of independent fusion events in different lineages also cannot be ignored.

Another important aspect of the analyses presented herein is the fact that, if taking into account the potential N-terminal extension on the *Ectocarpus siliculosus* protein, all TPI-GAPDH fusions examined possess putative mitochondrial targeting signals. Furthermore, our genome-based analyses showed that TPI-GAPDH-bearing stramenopiles (*Phaeodactylum tricornutum*, *Ectocarpus siliculosus*, *Phytophthora infestans* and *Blastocystis hominis*) possess apparent mitochondrial-localized enzymes for the latter half of the glycolytic pathway downstream of GAPDH (Table 2). This suggests that in these organisms at least some glycolytic reactions occur in the mitochondrion. Although these predictions are currently based entirely on *in silico* analyses, this prediction is feasible given that (a) a mitochondrial TPI-GAPDH has been confirmed by immuno-electron microscopy in *Phaeodactylum tricornutum* [7], and (b) these proteins are predicted to be mitochondrial-targeted with four different programs. In addition to these four stramenopiles, while *Aureococcus anophagefferens* and *Bigelowiella natans* apparently lack a TPI-GAPDH fusion, they nevertheless appear to have mitochondrion-localized glycolytic enzymes for three and two reactions in the latter half of the glycolytic pathway, respectively. Given that a growing number of enzymes involved in glycolysis have been reported to possess dual functions [52], [53], [54], [55], [56], we cannot rule out the possibility that the putative mitochondrial-targeted enzymes analyzed herein have non-glycolytic functions. However, glycolytic roles for those proteins seem likely when taking into account the fact that *Phaeodactylum tricornutum* and *Phytophthora infestans* have a strongly predicted, continuous set of mitochondrial-targeted enzymes corresponding to the latter half of glycolysis (Table 2). To reduce the likelihood of false-positive predictions, we combined results from multiple prediction programs. Nevertheless, results from a single prediction program can contain false-negatives and this number increases when multiple prediction results are combined [38], [39]. Consequently, it is likely that genuine mitochondrial-targeted enzymes have been missed by our stringent method. It is thus important to consider that negative mitochondrial targeting predictions obtained in this study are not necessarily evidence for non-mitochondrial localization. The subcellular predictions of the glycolytic enzymes examined herein should be confirmed experimentally using biochemical approaches such as proteomics.

Intriguingly, mitochondrion-associated glycolytic enzymes were found in proteomic analyses of land plants, yeast and the ciliate *Tetrahymena thermophila* [57], [58], [59], [60]. These instances are distinct from the mitochondrial-targeted glycolytic enzymes discussed in our study in that the enzymes identified by proteomics were deemed to be associated with the outer membrane of mitochondrion, not located inside the organelle (an exception is the enolase of *T. thermophila*, which possesses an obvious mitochondrial targeting signal [59]). Nonetheless, together with our results, these observations further support the notion of a close association between glycolytic enzymes and mitochondria across a broad spectrum of eukaryotic diversity. In *Arabidopsis thaliana*, it has been shown that the degree of association between glycolytic enzymes and the outer membrane of the mitochondrion correlates with the level of cellular respiration [60]. This situation is thought to regulate the channeling of pyruvate, the end product of glycolysis, into mitochondria to satisfy mitochondrial demand [60]. Although speculative, it is possible that the putative mitochondrial glycolytic enzymes detected in our study also play a role in modulating glycolytic flux and energy production, while the cytosolic enzymes are used for generating carbon skeletons involved in the biosynthesis of compounds such as amino acids and fatty acids. If true, transport mechanisms for relocating glycolytic intermediates into and out of mitochondria might play key regulatory roles. Candidate mitochondrial transporters for such intermediates have thus far not been identified.

Phylogenetic trees of PK and PGK showed monophyletic clades that were dominated by proteins predicted to be mitochondrial targeted (Figure 3 and Figure 4). The ‘mitochondrial’ PK clade also includes a *Bigelowiella natans* sequence with high bootstrap support. At least for PK, this is consistent with the possibility that the putative mitochondrial glycolytic enzymes from *Bigelowiella natans* and stramenopiles share a common origin. Although the support was low, the phylogenetic affinity between *Bigelowiella natans* and stramenopile sequences can also be seen in the PGAM tree (Figure S3). These results add further support to the existence of a phylogenetic relationship between the supergroups Rhizaria and stramenopiles beyond the TPI-GAPDH fusion.

A mitochondrial PK was previously reported from the apicomplexan parasite *Toxoplasma gondii* [61]. This PK was shown to be dual-targeted to both the ‘apicoplast’ (a relic plastid of secondary endosymbiotic origin [62]) and the mitochondrion. In spite of the resemblance in targeting, the *T. gondii* PK (BAG14336) did not branch with the ‘mitochondrial’ PK clade in our analysis (Figure 3), suggesting that the dual-targeted PK evolved independently.

It is widely accepted that modern-day mitochondria (and their derivatives) are descended from an alphaproteobacterium [63], [64], [65]. During the transition from endosymbiont to organelle, many genes on the endosymbiont chromosome were lost or relocated to the host nucleus. Many (but not all) of these genes now encode proteins that are targeted to the mitochondrion. Given that extant free-living alphaproteobacteria possess a glycolytic pathway, it is likely that the progenitor of mitochondria also possessed the pathway. However, there is no strong indication that glycolytic enzymes in present-day eukaryotic cells are of alphaproteobacterial ancestry [66] and glycolysis is widely considered to be a cytosolic pathway. Yet together with the existence of ‘glycolysis in the mitochondrion’ shown previously [5], [7], the results presented herein underscore the complexity of metabolic compartmentalization in protists, and emphasize the fact that knowledge of core metabolic pathways gleaned from studies of model animals and/or plants do not necessarily apply to eukaryotes as a whole.

## Supporting Information

Figure S1 **Maximum likelihood tree of GAPDH protein sequences with unstable sequences removed.** The *Thecamonas trahens* and *Bicosoeca* sp. sequences shown in the TPI-fused GAPDH clade were removed from the analysis shown in Figure 2. Phylogenetic methods and data presentation are as in Figure 2.
(PDF)

Figure S2 **Maximum likelihood tree of TPI protein sequences.** TPI ML tree constructed using RAxML and the LG+I+G+F model. TPI sequences fused with GAPDH are highlighted by colored boxes. Bootstrap values are as in Figure 2. The root was arbitrarily chosen. Scale bar shows the number of inferred amino acid substitutions per site.
(PDF)

Figure S3 **Maximum likelihood tree of PGAM protein sequences.** PGAM tree constructed using RAxML and the LG+G model. Putative mitochondrial-targeted PGAM sequences are highlighted by colored boxes. Presentation details are as in Figure 2.
(PDF)

Figure S4 **Maximum likelihood tree of enolase protein sequences.** Enolase tree constructed using RAxML with the LG+G+F model. Putative mitochondrial-targeted enolase sequences are highlighted by colored boxes. Details are as in Figure 2.
(PDF)

Table S1 Information for sequences used in phylogenetic analyses.
(PDF)

Table S2 TPI-GAPDH subcellular localization predictions.
(PDF)

Table S3 Subcellular localization predictions for various glycolytic enzymes.
(PDF)
