# Supplementary figures and images for: Broad Distribution of TPI-GAPDH Fusion Proteins among Eukaryotes: Evidence for Glycolytic Reactions in the Mitochondrion?
Source: PLoS One. 2012 Dec 20;7(12):e52340. doi: 10.1371/journal.pone.0052340 (PMC3527533; doi:10.1371/journal.pone.0052340)

# FIGURE S1

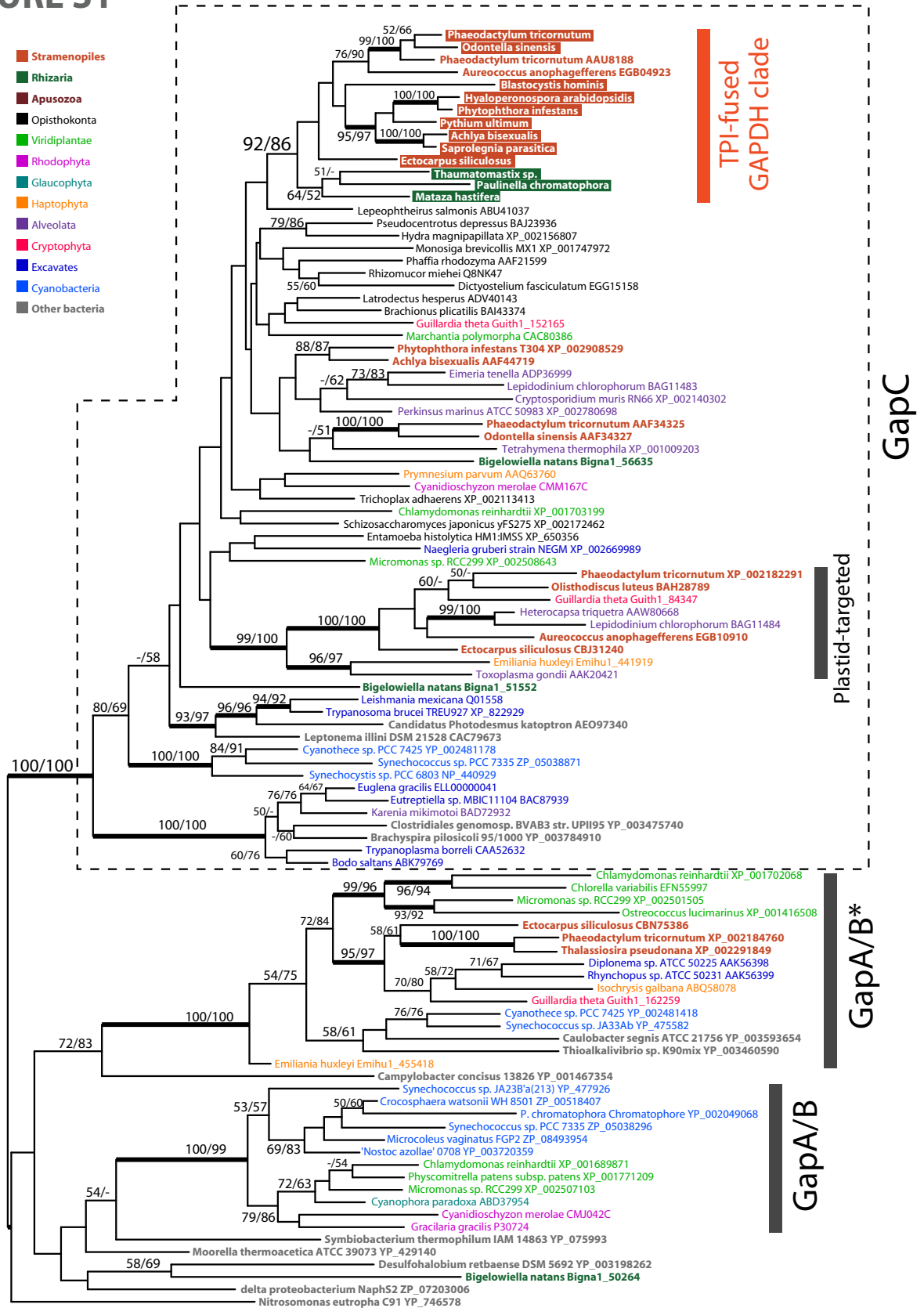

Supplement: Figure S1 — Maximum likelihood tree of GAPDH protein sequences with unstable sequences removed. The Thecamonas trahens and Bicosoeca sp. sequences shown in the TPI-fused GAPDH clade were removed from the analysis shown in Figure 2. Phylogenetic methods and data presentation are as in Figure 2. (PDF) [file pone.0052340.s001.pdf]

FIGURE S2

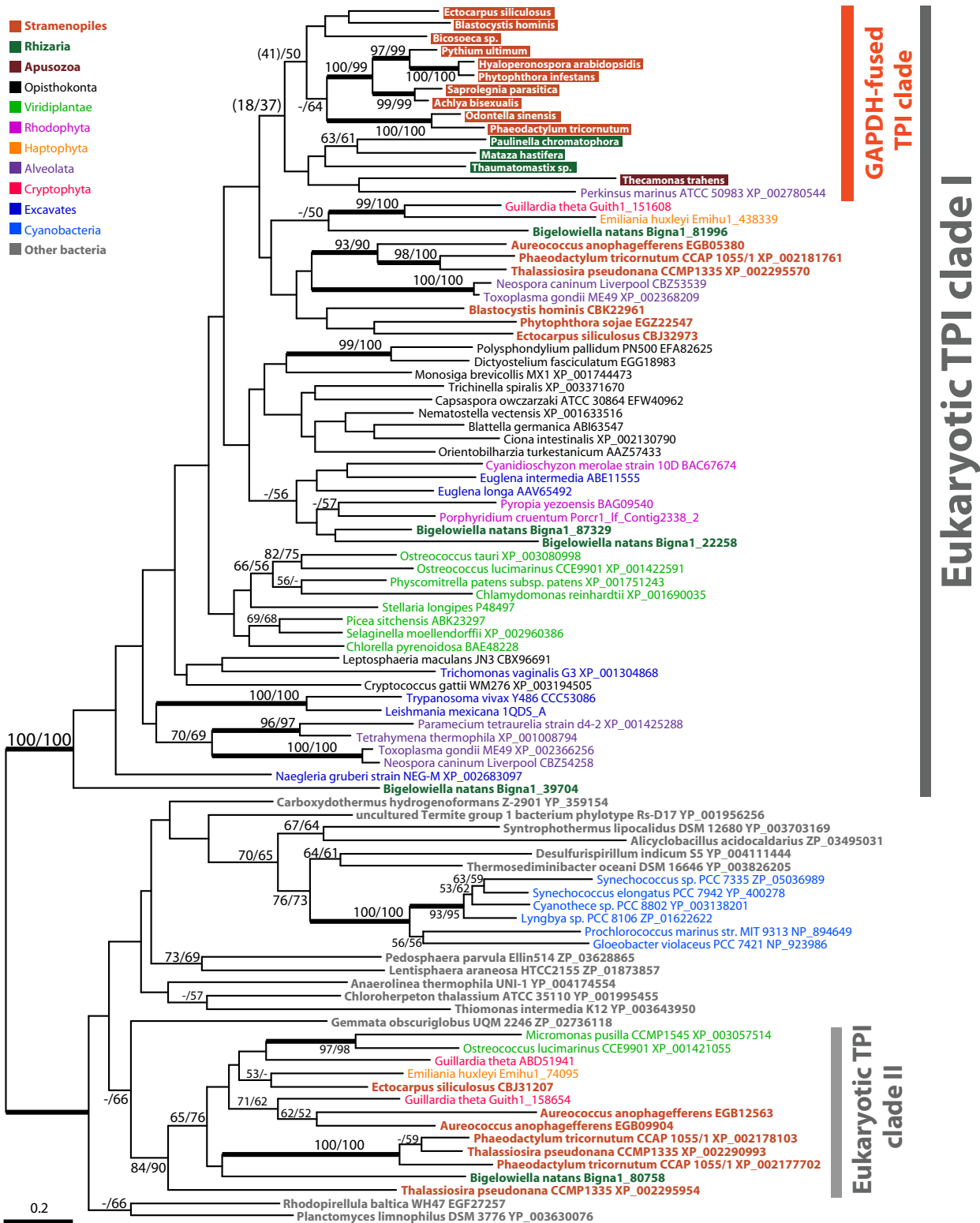

Supplement: Figure S2 — Maximum likelihood tree of TPI protein sequences. TPI ML tree constructed using RAxML and the LG+I+G+F model. TPI sequences fused with GAPDH are highlighted by colored boxes. Bootstrap values are as in Figure 2. The root was arbitrarily chosen. Scale bar shows the number of inferred amino acid substitutions per site. (PDF) [file pone.0052340.s002.pdf]

# FIGURE S4

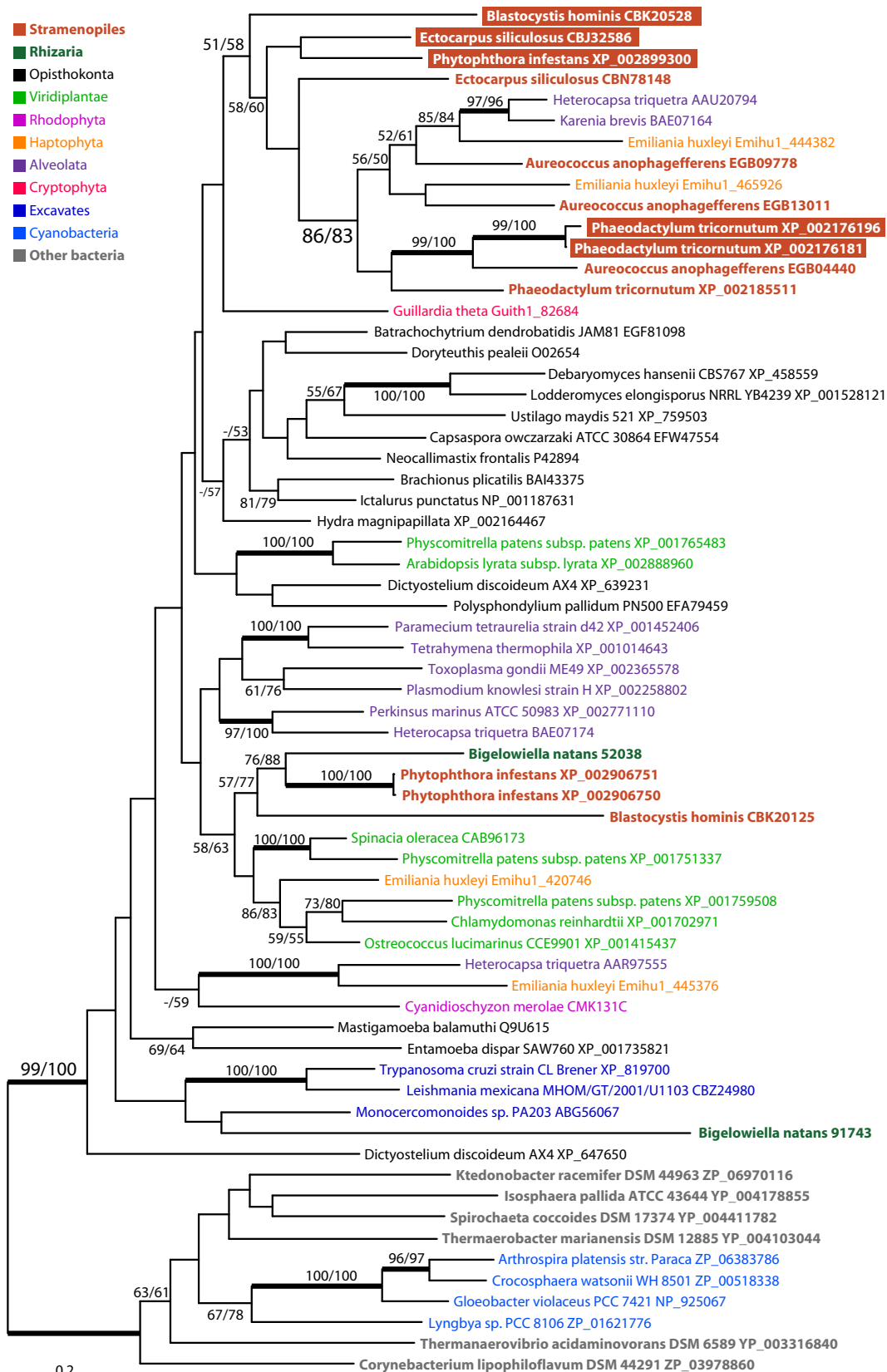

Eukaryotic enolase

Supplement: Figure S4 — Maximum likelihood tree of enolase protein sequences. Enolase tree constructed using RAxML with the LG+G+F model. Putative mitochondrial-targeted enolase sequences are highlighted by colored boxes. Details are as in Figure 2. (PDF) [file pone.0052340.s004.pdf]
