# Supplementary material for: Broad Distribution of TPI-GAPDH Fusion Proteins among Eukaryotes: Evidence for Glycolytic Reactions in the Mitochondrion?
Source: PLoS One. 2012 Dec 20;7(12):e52340. doi: 10.1371/journal.pone.0052340 (PMC3527533; doi:10.1371/journal.pone.0052340)
Supplement: Figure S3 — Maximum likelihood tree of PGAM protein sequences. PGAM tree constructed using RAxML and the LG+G model. Putative mitochondrial-targeted PGAM sequences are highlighted by colored boxes. Presentation details are as in Figure 2. (PDF) [file pone.0052340.s003.pdf]

FIGURE S3

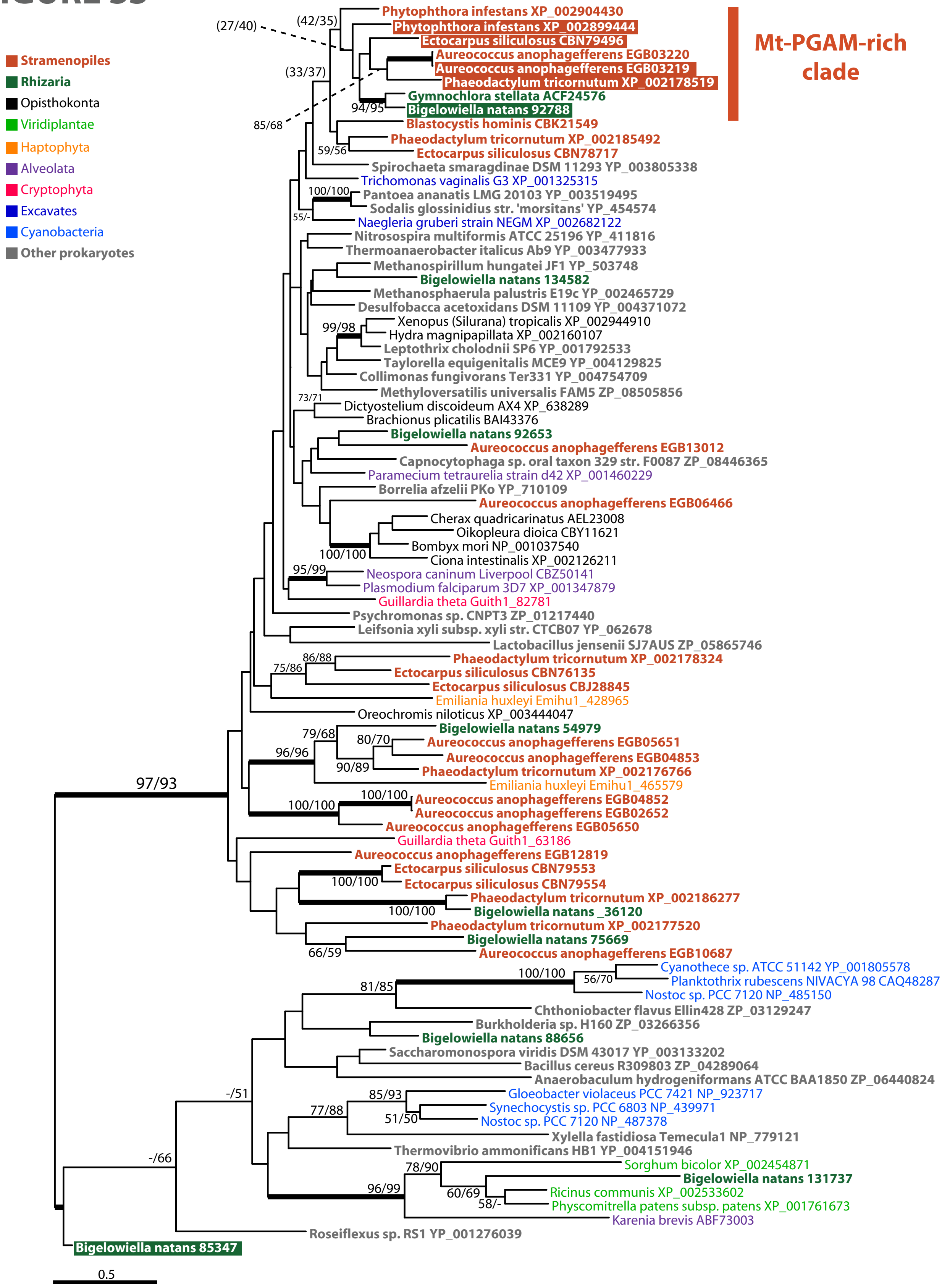

Stramenopiles
Rhizaria
Opisthokonta
Viridiplantae
Haptophyta
Alveolata
Cryptophyta
Excavates
Cyanobacteria
Other prokaryotes
Mt-PGAM-rich clade
(27/40)
(42/35)
(33/37)
85/68
94/95
59/56
Phytophthora infestans XP_002904430
Phytophthora infestans XP_002899444
Ectocarpus siliculosus CBN79496
Aureococcus anophagefferens EGB03220
Aureococcus anophagefferens EGB03219
Phaeodactylum tricornutum XP_002178519
Gymnochlora stellata ACF24576
Bigelowiella natans 92788
Blastocystis hominis CBK21549
Phaeodactylum tricornutum XP_002185492
Ectocarpus siliculosus CBN78717
Spirochaeta smaragdinae DSM 11293 YP_003805338
Trichomonas vaginalis G3 XP_001325315
100/100
Pantoea ananatis LMG 20103 YP_003519495
Sodalis glossinidius str. 'morsitans' YP_454574
55/-
Naegleria gruberi strain NEGM XP_002682122
Nitrosospira multiformis ATCC 25196 YP_411816
Thermoanaerobacter italicus Ab9 YP_003477933
Methanospirillum hungatei JF1 YP_503748
Bigelowiella natans 134582
Methanosphaerula palustris E19c YP_002465729
Desulfobacca acetoxidans DSM 11109 YP_004371072
99/98
Xenopus (Silurana) tropicalis XP_002944910
Hydra magnipapillata XP_002160107
Leptothrix cholodnii SP6 YP_001792533
Taylorella equigenitalis MCE9 YP_004129825
Collimonas fungivorans Ter331 YP_004754709
Methyloversatilis universalis FAM5 ZP_08505856
73/71
Dictyostelium discoideum AX4 XP_638289
Brachionus plicatilis BAI43376
Bigelowiella natans 92653
Aureococcus anophagefferens EGB13012
Capnocytophaga sp. oral taxon 329 str. F0087 ZP_08446365
Paramecium tetraurelia strain d42 XP_001460229
Borrelia afzelii PKo YP_710109
Aureococcus anophagefferens EGB06466
Cherax quadricarinatus AEL23008
Oikopleura dioica CBY11621
Bombyx mori NP_001037540
100/100
Ciona intestinalis XP_002126211
95/99
Neospora caninum Liverpool CBZ50141
Plasmodium falciparum 3D7 XP_001347879
Guillardia theta Guith1_82781
Psychromonas sp. CNPT3 ZP_01217440
Leifsonia xyli subsp. xyli str. CTCB07 YP_062678
Lactobacillus jensenii SJ7AUS ZP_05865746
86/88
75/86
Phaeodactylum tricornutum XP_002178324
Ectocarpus siliculosus CBN76135
Ectocarpus siliculosus CBJ28845
Emiliania huxleyi Emihu1_428965
Oreochromis niloticus XP_003444047
79/68
Bigelowiella natans 54979
80/70
Aureococcus anophagefferens EGB05651
96/96
Aureococcus anophagefferens EGB04853
90/89
Phaeodactylum tricornutum XP_002176766
Emiliania huxleyi Emihu1_465579
97/93
100/100
Aureococcus anophagefferens EGB04852
100/100
Aureococcus anophagefferens EGB02652
Aureococcus anophagefferens EGB05650
Guillardia theta Guith1_63186
Aureococcus anophagefferens EGB12819
Ectocarpus siliculosus CBN79553
100/100
Ectocarpus siliculosus CBN79554
Phaeodactylum tricornutum XP_002186277
100/100
Bigelowiella natans _36120
Phaeodactylum tricornutum XP_002177520
Bigelowiella natans 75669
66/59
Aureococcus anophagefferens EGB10687
100/100
Cyanothece sp. ATCC 51142 YP_001805578
56/70
Planktothrix rubescens NIVACYA 98 CAQ48287
81/85
Nostoc sp. PCC 7120 NP_485150
Chthoniobacter flavus Ellin428 ZP_03129247
Burkholderia sp. H160 ZP_03266356
Bigelowiella natans 88656
Saccharomonospora viridis DSM 43017 YP_003133202
Bacillus cereus R309803 ZP_04289064
Anaerobaculum hydrogeniformans ATCC BAA1850 ZP_06440824
-/51
85/93
Gloeobacter violaceus PCC 7421 NP_923717
77/88
Synechocystis sp. PCC 6803 NP_439971
51/50
Nostoc sp. PCC 7120 NP_487378
Xylella fastidiosa Temecula1 NP_779121
Thermovibrio ammonificans HB1 YP_004151946
-/66
78/90
Sorghum bicolor XP_002454871
Bigelowiella natans 131737
96/99
60/69
Ricinus communis XP_002533602
58/-
Physcomitrella patens subsp. patens XP_001761673
Karenia brevis ABF73003
Roseiflexus sp. RS1 YP_001276039
Bigelowiella natans 85347
0.5
