# Supplementary material for: Broad Distribution of TPI-GAPDH Fusion Proteins among Eukaryotes: Evidence for Glycolytic Reactions in the Mitochondrion?
Source: PLoS One. 2012 Dec 20;7(12):e52340. doi: 10.1371/journal.pone.0052340 (PMC3527533; doi:10.1371/journal.pone.0052340)
Supplement: Table S1 — Information for sequences used in phylogenetic analyses. (PDF) [file pone.0052340.s005.pdf]

Table S1. Information for sequences used in phylogenetic analyses

Enzyme	Organism	Domain	Group	Subgroup	GenBank accession numbers or other IDs	Note
GAPDH	Cryptosporidium muris RN66	Eukaryota	Alveolata	Apicomplexa	XP_002140302	
GAPDH	Eimeria tenella	Eukaryota	Alveolata	Apicomplexa	ADP36999	
GAPDH	Toxoplasma gondii	Eukaryota	Alveolata	Apicomplexa	AAK20421	
GAPDH	Tetrahymena thermophila	Eukaryota	Alveolata	Ciliophora	XP_001009203	
GAPDH	Heterocapsa triquetra	Eukaryota	Alveolata	Dinophyceae	AAW80668	
GAPDH	Karenia mikimotoi	Eukaryota	Alveolata	Dinophyceae	BAD72932	
GAPDH	Lepidodinium chlorophorum	Eukaryota	Alveolata	Dinophyceae	BAG11483	
GAPDH	Lepidodinium chlorophorum	Eukaryota	Alveolata	Dinophyceae	BAG11484	
GAPDH	Perkinsus marinus ATCC 50983	Eukaryota	Alveolata	Perkinsea	XP_002780698	
GAPDH	Entamoeba histolytica HM1:IMSS	Eukaryota	Amoebozoa	Archamoebae	XP_650356	
GAPDH	Dictyostelium fasciculatum	Eukaryota	Amoebozoa	Mycetozoa	EGG15158	
GAPDH	Thecamonas trahens	Eukaryota	Apusozoa	Apusomonadidae	See table S2	TPI-fused
GAPDH	Monosiga brevicollis MX1	Eukaryota	Choanoflagellida	Codonosigidae	XP_001747972	
GAPDH	Guillardia theta	Eukaryota	Cryptophyta	Pyrenomonadales	Guith1_152165	retrieved from [1]
GAPDH	Guillardia theta	Eukaryota	Cryptophyta	Pyrenomonadales	Guith1_84347	retrieved from [1]
GAPDH	Guillardia theta	Eukaryota	Cryptophyta	Pyrenomonadales	Guith1_162259	retrieved from [1]
GAPDH	Diplonema sp. ATCC 50225	Eukaryota	Euglenozoa	Diplonemida	AAK56398	
GAPDH	Rhynchopus sp. ATCC 50231	Eukaryota	Euglenozoa	Diplonemida	AAK56399	
GAPDH	Euglena gracilis	Eukaryota	Euglenozoa	Euglenida	ELL00000041	retrieved from [2]
GAPDH	Eutreptiella sp. MBIC11104	Eukaryota	Euglenozoa	Euglenida	BAC87939	
GAPDH	Bodo saltans	Eukaryota	Euglenozoa	Kinetoplastida	ABK79769	
GAPDH	Leishmania mexicana	Eukaryota	Euglenozoa	Kinetoplastida	Q01558	
GAPDH	Trypanoplasma borreli	Eukaryota	Euglenozoa	Kinetoplastida	CAA52632	
GAPDH	Trypanosoma brucei TREU927	Eukaryota	Euglenozoa	Kinetoplastida	XP_822929	
GAPDH	Phaffia rhodozyma	Eukaryota	Fungi	Dikarya	AAF21599	
GAPDH	Schizosaccharomyces japonicus yFS275	Eukaryota	Fungi	Dikarya	XP_002172462	
GAPDH	Rhizomucor miehei	Eukaryota	Fungi	Fungi incertae sedis	Q8NK47	
GAPDH	Cyanophora paradoxa	Eukaryota	Glaucocystophyceae	Cyanophoraceae	ABD37954	
GAPDH	Emiliania huxleyi	Eukaryota	Haptophyceae	Isochrysidales	Emihu1_441919	retrieved from [3]
GAPDH	Emiliania huxleyi	Eukaryota	Haptophyceae	Isochrysidales	Emihu1_455418	retrieved from [3]
GAPDH	Isochrysis galbana	Eukaryota	Haptophyceae	Isochrysidales	ABQ58078	
GAPDH	Prymnesium parvum	Eukaryota	Haptophyceae	Prymnesiales	AAQ63760	
GAPDH	Naegleria gruberi strain NEGM	Eukaryota	Heterolobosea	Schizopyrenida	XP_002669989	
GAPDH	Latrodectus hesperus	Eukaryota	Metazoa	Arthropoda	ADV40143	
GAPDH	Lepeophtheirus salmonis	Eukaryota	Metazoa	Arthropoda	ABU41037	
GAPDH	Hydra magnipapillata	Eukaryota	Metazoa	Cnidaria	XP_002156807	
GAPDH	Pseudocentrotus depressus	Eukaryota	Metazoa	Echinodermata	BAJ23936	
GAPDH	Trichoplax adhaerens	Eukaryota	Metazoa	Placozoa	XP_002113413	
GAPDH	Brachionus plicatilis	Eukaryota	Metazoa	Rotifera	BAI43374	
GAPDH	Bigelowiella natans	Eukaryota	Rhizaria	Cercozoa	Bigna1_50264	retrieved from [4]
GAPDH	Bigelowiella natans	Eukaryota	Rhizaria	Cercozoa	Bigna1_51552	retrieved from [4]
GAPDH	Bigelowiella natans	Eukaryota	Rhizaria	Cercozoa	Bigna1_56635	retrieved from [4]
GAPDH	Mataza hastifera	Eukaryota	Rhizaria	Cercozoa	See table S2	TPI-fused
GAPDH	Paulinella chromatophora	Eukaryota	Rhizaria	Cercozoa	See table S2	TPI-fused
GAPDH	Paulinella chromatophora	Eukaryota	Rhizaria	Cercozoa	YP_002049068	
GAPDH	Thaumatomastix sp.	Eukaryota	Rhizaria	Cercozoa	See table S2	TPI-fused
GAPDH	Cyanidioschyzon merolae	Eukaryota	Rhodophyta	Bangiophyceae	CMM167C	retrieved from [5]
GAPDH	Cyanidioschyzon merolae	Eukaryota	Rhodophyta	Bangiophyceae	CMJ042C	retrieved from [5]
GAPDH	Gracilaria gracilis	Eukaryota	Rhodophyta	Florideophyceae	P30724	
GAPDH	Odontella sinensis	Eukaryota	Stramenopile	Bacillariophyta	See table S2	TPI-fused
GAPDH	Odontella sinensis	Eukaryota	Stramenopile	Bacillariophyta	AAF34327	
GAPDH	Phaeodactylum tricornutum	Eukaryota	Stramenopile	Bacillariophyta	AAU81889	
GAPDH	Phaeodactylum tricornutum	Eukaryota	Stramenopile	Bacillariophyta	See table S2	TPI-fused
GAPDH	Phaeodactylum tricornutum	Eukaryota	Stramenopile	Bacillariophyta	AAF34325	
GAPDH	Phaeodactylum tricornutum CCAP 1055/1	Eukaryota	Stramenopile	Bacillariophyta	XP_002182291	
GAPDH	Phaeodactylum tricornutum CCAP 1055/1	Eukaryota	Stramenopile	Bacillariophyta	XP_002184760	
GAPDH	Thalassiosira pseudonana CCMP1335	Eukaryota	Stramenopile	Bacillariophyta	XP_002291849	
GAPDH	Bicosoeca sp.	Eukaryota	Stramenopile	Bicosoecea	See table S2	TPI-fused
GAPDH	Blastocystis hominis	Eukaryota	Stramenopile	Blastocystae	See table S2	TPI-fused
GAPDH	Olisthodiscus luteus	Eukaryota	Stramenopile	Olisthodiscus	BAH28789	
GAPDH	Achlya bisexualis	Eukaryota	Stramenopile	Oomycetes	See table S2	TPI-fused
GAPDH	Achlya bisexualis	Eukaryota	Stramenopile	Oomycetes	AAF44719	
GAPDH	Hyaloperonospora arabidopsidis	Eukaryota	Stramenopile	Oomycetes	See table S2	TPI-fused
GAPDH	Phytophthora infestans	Eukaryota	Stramenopile	Oomycetes	See table S2	TPI-fused
GAPDH	Phytophthora infestans T304	Eukaryota	Stramenopile	Oomycetes	XP_002908529	
GAPDH	Pythium ultimum	Eukaryota	Stramenopile	Oomycetes	See table S2	TPI-fused
GAPDH	Saprolegnia parasitica	Eukaryota	Stramenopile	Oomycetes	See table S2	TPI-fused
GAPDH	Aureococcus anophagefferens	Eukaryota	Stramenopile	Pelagophyceae	EGB04923	
GAPDH	Aureococcus anophagefferens	Eukaryota	Stramenopile	Pelagophyceae	EGB10910	
GAPDH	Ectocarpus siliculosus	Eukaryota	Stramenopile	PX clade	See table S2	TPI-fused
GAPDH	Ectocarpus siliculosus	Eukaryota	Stramenopile	PX clade	CBJ31240	
GAPDH	Ectocarpus siliculosus	Eukaryota	Stramenopile	PX clade	CBN75386	
GAPDH	Chlamydomonas reinhardtii	Eukaryota	Viridiplantae	Chlorophyta	XP_001703199	
GAPDH	Chlamydomonas reinhardtii	Eukaryota	Viridiplantae	Chlorophyta	XP_001689871	
GAPDH	Chlamydomonas reinhardtii	Eukaryota	Viridiplantae	Chlorophyta	XP_001702068	
GAPDH	Chlorella variabilis	Eukaryota	Viridiplantae	Chlorophyta	EFN55997	

GAPDH	Micromonas sp. RCC299	Eukaryota	Viridiplantae	Chlorophyta	XP_002508643	
GAPDH	Micromonas sp. RCC299	Eukaryota	Viridiplantae	Chlorophyta	XP_002507103	
GAPDH	Micromonas sp. RCC299	Eukaryota	Viridiplantae	Chlorophyta	XP_002501505	
GAPDH	Ostreococcus lucimarinus CCE9901	Eukaryota	Viridiplantae	Chlorophyta	XP_001416508	
GAPDH	Marchantia polymorpha	Eukaryota	Viridiplantae	Streptophyta	CAC80386	
GAPDH	Physcomitrella patens subsp. patens	Eukaryota	Viridiplantae	Streptophyta	XP_001771209	
GAPDH	Crocosphaera watsonii WH 8501	Bacteria	Cyanobacteria	Chroococcales	ZP_00518407	
GAPDH	Cyanothece sp. PCC 7425	Bacteria	Cyanobacteria	Chroococcales	YP_002481178	
GAPDH	Cyanothece sp. PCC 7425	Bacteria	Cyanobacteria	Chroococcales	YP_002481418	
GAPDH	Synechococcus sp. JA23B'a(213)	Bacteria	Cyanobacteria	Chroococcales	YP_477926	
GAPDH	Synechococcus sp. JA33Ab	Bacteria	Cyanobacteria	Chroococcales	YP_475582	
GAPDH	Synechococcus sp. PCC 7335	Bacteria	Cyanobacteria	Chroococcales	ZP_05038871	
GAPDH	Synechococcus sp. PCC 7335	Bacteria	Cyanobacteria	Chroococcales	ZP_05038296	
GAPDH	Synechocystis sp. PCC 6803	Bacteria	Cyanobacteria	Chroococcales	NP_440929	
GAPDH	'Nostoc azollae' 0708	Bacteria	Cyanobacteria	Nostocales	YP_003720359	
GAPDH	Microcoleus vaginatus FGP2	Bacteria	Cyanobacteria	Oscillatoriales	ZP_08493954	
GAPDH	Clostridiales genomosp. BVAB3 str. UPII95	Bacteria	Firmicutes	Clostridia	YP_003475740	
GAPDH	Moorella thermoacetica ATCC 39073	Bacteria	Firmicutes	Clostridia	YP_429140	
GAPDH	Symbiobacterium thermophilum IAM 14863	Bacteria	Firmicutes	Clostridia	YP_075993	
GAPDH	Caulobacter segnis ATCC 21756	Bacteria	Proteobacteria	Alphaproteobacteria	YP_003593654	
GAPDH	Nitrosomonas eutropha C91	Bacteria	Proteobacteria	Betaproteobacteria	YP_746578	
GAPDH	Desulfohalobium retbaense DSM 5692	Bacteria	Proteobacteria	Deltaproteobacteria	YP_003198262	
GAPDH	delta proteobacterium NaphS2	Bacteria	Proteobacteria	Deltaproteobacteria	ZP_07203006	
GAPDH	Campylobacter concisus 13826	Bacteria	Proteobacteria	Epsilonproteobacteria	YP_001467354	
GAPDH	Candidatus Photodesmus katoptron	Bacteria	Proteobacteria	Gammaproteobacteria	AEO97340	
GAPDH	Thioalkalivibrio sp. K90mix	Bacteria	Proteobacteria	Gammaproteobacteria	YP_003460590	
GAPDH	Brachyspira pilosicoli 95/1000	Bacteria	Spirochaetes	Spirochaetales	YP_003784910	
GAPDH	Leptonema illini DSM 21528	Bacteria	Spirochaetes	Spirochaetales	CAC79673	
TPI	Neospora caninum Liverpool	Eukaryota	Alveolata	Apicomplexa	CBZ53539	
TPI	Neospora caninum Liverpool	Eukaryota	Alveolata	Apicomplexa	CBZ54258	
TPI	Toxoplasma gondii ME49	Eukaryota	Alveolata	Apicomplexa	XP_002368209	
TPI	Toxoplasma gondii ME49	Eukaryota	Alveolata	Apicomplexa	XP_002366256	
TPI	Paramecium tetraurelia strain d4-2	Eukaryota	Alveolata	Ciliophora	XP_001425288	
TPI	Tetrahymena thermophila	Eukaryota	Alveolata	Ciliophora	XP_001008794	
TPI	Perkinsus marinus ATCC 50983	Eukaryota	Alveolata	Perkinsea	XP_002780544	
TPI	Dictyostelium fasciculatum	Eukaryota	Amoebozoa	Mycetozoa	EGG18983	
TPI	Polysphondylium pallidum PN500	Eukaryota	Amoebozoa	Mycetozoa	EFA82625	
TPI	Thecamonas trahens	Eukaryota	Apusozoa	Apusomonadidae	See table S2	GAPDH-fused
TPI	Monosiga brevicollis MX1	Eukaryota	Choanoflagellida	Codonosigidae	XP_001744473	
TPI	Guillardia theta	Eukaryota	Cryptophyta	Pyrenomonadales	Guith1_151608	retrieved from [1]
TPI	Guillardia theta	Eukaryota	Cryptophyta	Pyrenomonadales	Guith1_158654	retrieved from [1]
TPI	Guillardia theta	Eukaryota	Cryptophyta	Pyrenomonadales	ABD51941	
TPI	Euglena intermedia	Eukaryota	Euglenozoa	Euglenida	ABE11555	
TPI	Euglena longa	Eukaryota	Euglenozoa	Euglenida	AAV65492	
TPI	Leishmania mexicana	Eukaryota	Euglenozoa	Kinetoplastida	1QDS_A	
TPI	Trypanosoma vivax Y486	Eukaryota	Euglenozoa	Kinetoplastida	CCC53086	
TPI	Cryptococcus gattii WM276	Eukaryota	Fungi	Dikarya	XP_003194505	
TPI	Leptosphaeria maculans JN3	Eukaryota	Fungi	Dikarya	CBX96691	
TPI	Emiliania huxleyi	Eukaryota	Haptophyceae	Isochrysidales	Emihu1_438339	retrieved from [3]
TPI	Emiliania huxleyi	Eukaryota	Haptophyceae	Isochrysidales	Emihu1_74095	retrieved from [3]
TPI	Naegleria gruberi strain NEG-M	Eukaryota	Heterolobosea	Schizopyrenida	XP_002683097	
TPI	Capsaspora owczarzaki ATCC 30864	Eukaryota	Ichthyosporea	Capsaspora	EFW40962	
TPI	Blattella germanica	Eukaryota	Metazoa	Arthropoda	ABI63547	
TPI	Ciona intestinalis	Eukaryota	Metazoa	Chordata	XP_002130790	
TPI	Nematostella vectensis	Eukaryota	Metazoa	Cnidaria	XP_001633516	
TPI	Trichinella spiralis	Eukaryota	Metazoa	Nematoda	XP_003371670	
TPI	Orientobilharzia turkestanicum	Eukaryota	Metazoa	Platyhelminthes	AAZ57433	
TPI	Trichomonas vaginalis G3	Eukaryota	Parabasalia	Trichomonadida	XP_001304868	
TPI	Bigelowiella natans	Eukaryota	Rhizaria	Cercozoa	Bigna1_87329	retrieved from [4]
TPI	Bigelowiella natans	Eukaryota	Rhizaria	Cercozoa	Bigna1_22258	retrieved from [4]
TPI	Bigelowiella natans	Eukaryota	Rhizaria	Cercozoa	Bigna1_81996	retrieved from [4]
TPI	Bigelowiella natans	Eukaryota	Rhizaria	Cercozoa	Bigna1_39704	retrieved from [4]
TPI	Bigelowiella natans	Eukaryota	Rhizaria	Cercozoa	Bigna1_80758	retrieved from [4]
TPI	Mataza hastifera	Eukaryota	Rhizaria	Cercozoa	See table S2	GAPDH-fused
TPI	Paulinella chromatophora	Eukaryota	Rhizaria	Cercozoa	See table S2	GAPDH-fused
TPI	Thaumatomastix sp.	Eukaryota	Rhizaria	Cercozoa	See table S2	GAPDH-fused
TPI	Cyanidioschyzon merolae strain 10D	Eukaryota	Rhodophyta	Bangiophyceae	BAC67674	
TPI	Porphyridium cruentum	Eukaryota	Rhodophyta	Bangiophyceae	Porcr1_lf_Contig2338_2	retrieved from [6]
TPI	Pyropia yezoensis	Eukaryota	Rhodophyta	Bangiophyceae	BAG09540	
TPI	Odontella sinensis	Eukaryota	Stramenopile	Bacillariophyta	See table S2	GAPDH-fused
TPI	Phaeodactylum tricornutum	Eukaryota	Stramenopile	Bacillariophyta	See table S2	GAPDH-fused
TPI	Phaeodactylum tricornutum CCAP 1055/1	Eukaryota	Stramenopile	Bacillariophyta	XP_002181761	
TPI	Phaeodactylum tricornutum CCAP 1055/1	Eukaryota	Stramenopile	Bacillariophyta	XP_002178103	
TPI	Phaeodactylum tricornutum CCAP 1055/1	Eukaryota	Stramenopile	Bacillariophyta	XP_002177702	
TPI	Thalassiosira pseudonana CCMP1335	Eukaryota	Stramenopile	Bacillariophyta	XP_002295570	
TPI	Thalassiosira pseudonana CCMP1335	Eukaryota	Stramenopile	Bacillariophyta	XP_002290993	
TPI	Thalassiosira pseudonana CCMP1335	Eukaryota	Stramenopile	Bacillariophyta	XP_002295954	

TPI	Bicosoeca sp.	Eukaryota	Stramenopile	Bicosoecea	See table S2	GAPDH-fused
TPI	Blastocystis hominis	Eukaryota	Stramenopile	Blastocystae	See table S2	GAPDH-fused
TPI	Blastocystis hominis	Eukaryota	Stramenopile	Blastocystae	CBK22961	
TPI	Achlya bisexualis	Eukaryota	Stramenopile	Oomycetes	See table S2	GAPDH-fused
TPI	Hyaloperonospora arabidopsidis	Eukaryota	Stramenopile	Oomycetes	See table S2	GAPDH-fused
TPI	Phytophthora infestans	Eukaryota	Stramenopile	Oomycetes	See table S2	GAPDH-fused
TPI	Phytophthora sojae	Eukaryota	Stramenopile	Oomycetes	EGZ22547	
TPI	Pythium ultimum	Eukaryota	Stramenopile	Oomycetes	See table S2	GAPDH-fused
TPI	Saprolegnia parasitica	Eukaryota	Stramenopile	Oomycetes	See table S2	GAPDH-fused
TPI	Aureococcus anophagefferens	Eukaryota	Stramenopile	Pelagophyceae	EGB05380	
TPI	Aureococcus anophagefferens	Eukaryota	Stramenopile	Pelagophyceae	EGB09904	
TPI	Aureococcus anophagefferens	Eukaryota	Stramenopile	Pelagophyceae	EGB12563	
TPI	Ectocarpus siliculosus	Eukaryota	Stramenopile	PX clade	See table S2	GAPDH-fused
TPI	Ectocarpus siliculosus	Eukaryota	Stramenopile	PX clade	CBJ32973	
TPI	Ectocarpus siliculosus	Eukaryota	Stramenopile	PX clade	CBJ31207	
TPI	Chlamydomonas reinhardtii	Eukaryota	Viridiplantae	Chlorophyta	XP_001690035	
TPI	Chlorella pyrenoidosa	Eukaryota	Viridiplantae	Chlorophyta	BAE48228	
TPI	Micromonas pusilla CCMP1545	Eukaryota	Viridiplantae	Chlorophyta	XP_003057514	
TPI	Ostreococcus lucimarinus CCE9901	Eukaryota	Viridiplantae	Chlorophyta	XP_001422591	
TPI	Ostreococcus lucimarinus CCE9901	Eukaryota	Viridiplantae	Chlorophyta	XP_001421055	
TPI	Ostreococcus tauri	Eukaryota	Viridiplantae	Chlorophyta	XP_003080998	
TPI	Physcomitrella patens subsp. patens	Eukaryota	Viridiplantae	Streptophyta	XP_001751243	
TPI	Picea sitchensis	Eukaryota	Viridiplantae	Streptophyta	ABK23297	
TPI	Selaginella moellendorffii	Eukaryota	Viridiplantae	Streptophyta	XP_002960386	
TPI	Stellaria longipes	Eukaryota	Viridiplantae	Streptophyta	P48497	
TPI	Chloroherpeton thalassium ATCC 35110	Bacteria	Chlorobi	Chlorobia	YP_001995455	
TPI	Anaerolinea thermophila UNI-1	Bacteria	Chloroflexi	Anaerolineae	YP_004174554	
TPI	Desulfurispirillum indicum S5	Bacteria	Chrysiogenetes	Chrysiogenales	YP_004111444	
TPI	Cyanothece sp. PCC 8802	Bacteria	Cyanobacteria	Chroococcales	YP_003138201	
TPI	Synechococcus elongatus PCC 7942	Bacteria	Cyanobacteria	Chroococcales	YP_400278	
TPI	Synechococcus sp. PCC 7335	Bacteria	Cyanobacteria	Chroococcales	ZP_05036989	
TPI	Gloeobacter violaceus PCC 7421	Bacteria	Cyanobacteria	Gloeobacteria	NP_923986	
TPI	Lyngbya sp. PCC 8106	Bacteria	Cyanobacteria	Oscillatoriales	ZP_01622622	
TPI	Prochlorococcus marinus str. MIT 9313	Bacteria	Cyanobacteria	Prochlorales	NP_894649	
TPI	uncultured Termite group 1 bacterium phylotype Rs-D17	Bacteria	Elusimicrobia	environmental samples	YP_001956256	
TPI	Alicyclobacillus acidocaldarius LAA1	Bacteria	Firmicutes	Bacillales	ZP_03495031	
TPI	Carboxydothermus hydrogenoformans Z-2901	Bacteria	Firmicutes	Clostridia	YP_359154	
TPI	Syntrophothermus lipocalidus DSM 12680	Bacteria	Firmicutes	Clostridia	YP_003703169	
TPI	Thermosediminibacter oceani DSM 16646	Bacteria	Firmicutes	Clostridia	YP_003826205	
TPI	Lentisphaera araneosa HTCC2155	Bacteria	Lentisphaerae	Lentisphaerales	ZP_01873857	
TPI	Gemmata obscuriglobus UQM 2246	Bacteria	Planctomycetes	Planctomycetacia	ZP_02736118	
TPI	Planctomyces limnophilus DSM 3776	Bacteria	Planctomycetes	Planctomycetacia	YP_003630076	
TPI	Rhodopirellula baltica WH47	Bacteria	Planctomycetes	Planctomycetacia	EGF27257	
TPI	Thiomonas intermedia K12	Bacteria	Proteobacteria	Betaproteobacteria	YP_003643950	
TPI	Pedosphaera parvula Ellin514	Bacteria	Verrucomicrobia	Verrucomicrobiae	ZP_03628865	
PGK	Cryptosporidium muris RN66	Eukaryota	Alveolata	Apicomplexa	XP_002141250	
PGK	Theileria parva strain Muguga	Eukaryota	Alveolata	Apicomplexa	XP_766486	
PGK	Toxoplasma gondii ME49	Eukaryota	Alveolata	Apicomplexa	XP_002369762	
PGK	Moneuplotes crassus	Eukaryota	Alveolata	Ciliophora	O02608	
PGK	Paramecium tetraurelia strain d42	Eukaryota	Alveolata	Ciliophora	XP_001427410	
PGK	Tetrahymena thermophila	Eukaryota	Alveolata	Ciliophora	XP_001025708	
PGK	Heterocapsa triquetra	Eukaryota	Alveolata	Dinophyceae	AAW79324	
PGK	Perkinsus marinus ATCC 50983	Eukaryota	Alveolata	Perkinsea	XP_002767391	
PGK	Dictyostelium discoideum AX4	Eukaryota	Amoebozoa	Mycetozoa	XP_637130	
PGK	Polysphondylium pallidum PN500	Eukaryota	Amoebozoa	Mycetozoa	EFA79539	
PGK	Monosiga brevicollis MX1	Eukaryota	Choanoflagellida	Codonosigidae	XP_001745299	
PGK	Guillardia theta	Eukaryota	Cryptophyta	Pyrenomonadales	Guith1_80135	retrieved from [1]
PGK	Guillardia theta	Eukaryota	Cryptophyta	Pyrenomonadales	Guith1_149840	retrieved from [1]
PGK	Guillardia theta	Eukaryota	Cryptophyta	Pyrenomonadales	Guith1_91943	retrieved from [1]
PGK	Giardia intestinalis ATCC 50581	Eukaryota	Diplomonadida	Hexamitidae	EES98813	
PGK	Euglena gracilis	Eukaryota	Euglenozoa	Euglenida	ELL00002262	retrieved from [2]
PGK	Mycosphaerella graminicola IPO323	Eukaryota	Fungi	Dikarya	EGP91798	
PGK	Schizosaccharomyces pombe 972h	Eukaryota	Fungi	Dikarya	NP_596730	
PGK	Verticillium alboatrum VaMs.102	Eukaryota	Fungi	Dikarya	XP_003009759	
PGK	Yarrowia lipolytica CLIB122	Eukaryota	Fungi	Dikarya	P29407	
PGK	Rhizopus oryzae	Eukaryota	Fungi	Fungi incertae sedis	ABB88570	
PGK	Glomus mosseae	Eukaryota	Fungi	Glomeromycota	O74233	
PGK	Emiliania huxleyi	Eukaryota	Haptophyceae	Isochrysidales	Emihu1_365175	retrieved from [3]
PGK	Emiliania huxleyi	Eukaryota	Haptophyceae	Isochrysidales	Emihu1_72672	retrieved from [3]
PGK	Isochrysis galbana	Eukaryota	Haptophyceae	Isochrysidales	AAW79325	
PGK	Pavlova lutheri	Eukaryota	Haptophyceae	Pavlovales	AAW79327	
PGK	Naegleria gruberi	Eukaryota	Heterolobosea	Schizopyrenida	ABK81119	
PGK	Capsaspora owczarzaki ATCC 30864	Eukaryota	Ichthyosporea	Capsaspora	EFW45319	
PGK	Daphnia pulex	Eukaryota	Metazoa	Arthropoda	EFX90443	
PGK	Ixodes scapularis	Eukaryota	Metazoa	Arthropoda	XP_002407168	
PGK	Homo sapiens	Eukaryota	Metazoa	Chordata	2Y3I_A	
PGK	Monodelphis domestica	Eukaryota	Metazoa	Chordata	XP_001377109	

PGK	Hydra magnipapillata	Eukaryota	Metazoa	Cnidaria	XP_002156684	
PGK	Loa loa	Eukaryota	Metazoa	Nematoda	XP_003141782	
PGK	Opisthorchis sinensis	Eukaryota	Metazoa	Platyhelminthes	P50311	
PGK	Trichomonas vaginalis G3	Eukaryota	Parabasalia	Trichomonadida	XP_001314248	
PGK	Bigelowiella natans	Eukaryota	Rhizaria	Cercozoa	Bigna1_92275	retrieved from [4]
PGK	Bigelowiella natans	Eukaryota	Rhizaria	Cercozoa	Bigna1_92730	retrieved from [4]
PGK	Cyanidioschyzon merolae	Eukaryota	Rhodophyta	Bangiophyceae	BAD36768	
PGK	Porphyridium cruentum	Eukaryota	Rhodophyta	Bangiophyceae	Porcr1_lf_Contig7029_3	retrieved from [6]
PGK	Pyropia yezoensis	Eukaryota	Rhodophyta	Bangiophyceae	BAG09537	
PGK	Chondrus crispus	Eukaryota	Rhodophyta	Florideophyceae	AAK40345	
PGK	Chondrus crispus	Eukaryota	Rhodophyta	Florideophyceae	AAK40346	
PGK	Phaeodactylum tricornutum	Eukaryota	Stramenopile	Bacillariophyta	XP_002183701.1	
PGK	Phaeodactylum tricornutum	Eukaryota	Stramenopile	Bacillariophyta	XP_002182409.1	
PGK	Phaeodactylum tricornutum	Eukaryota	Stramenopile	Bacillariophyta	XP_002182724.1	
PGK	Blastocystis hominis	Eukaryota	Stramenopile	Blastocystae	CBK20833.2	
PGK	Phytophthora infestans	Eukaryota	Stramenopile	Oomycetes	XP_002908759.1	
PGK	Aureococcus anophagefferens	Eukaryota	Stramenopile	Pelagophyceae	EGB10290.1	
PGK	Aureococcus anophagefferens	Eukaryota	Stramenopile	Pelagophyceae	EGB09450.1	
PGK	Ectocarpus siliculosus	Eukaryota	Stramenopile	PX clade	CBN73855.1	
PGK	Ectocarpus siliculosus	Eukaryota	Stramenopile	PX clade	CBN75134.1	
PGK	Ectocarpus siliculosus	Eukaryota	Stramenopile	PX clade	CBN75135.1	
PGK	Ectocarpus siliculosus	Eukaryota	Stramenopile	PX clade	CBN79775.1	
PGK	Ectocarpus siliculosus	Eukaryota	Stramenopile	PX clade	CBN75623.1	
PGK	Chlorella variabilis	Eukaryota	Viridiplantae	Chlorophyta	EFN59488	
PGK	Chlorella variabilis	Eukaryota	Viridiplantae	Chlorophyta	EFN59289	
PGK	Micromonas sp. RCC299	Eukaryota	Viridiplantae	Chlorophyta	XP_002500825	
PGK	Volvox carteri f. nagariensis	Eukaryota	Viridiplantae	Chlorophyta	XP_002951648	
PGK	Physcomitrella patens subsp. patens	Eukaryota	Viridiplantae	Streptophyta	XP_001777571	
PGK	Atopobium vaginae PB189T14	Bacteria	Actinobacteria	Coriobacteridae	ZP_07318997	
PGK	Chloroherpeton thalassium ATCC 35110	Bacteria	Chlorobi	Chlorobia	YP_001995699	
PGK	Anaerolinea thermophila UNI1	Bacteria	Chloroflexi	Anaerolineae	YP_004173956	
PGK	Cyanothece sp. CCY0110	Bacteria	Cyanobacteria	Chroococcales	ZP_01729409	
PGK	Cyanothece sp. PCC 7425	Bacteria	Cyanobacteria	Chroococcales	YP_002481705	
PGK	Cyanothece sp. PCC 8801	Bacteria	Cyanobacteria	Chroococcales	YP_002374433	
PGK	Synechococcus elongatus PCC 7942	Bacteria	Cyanobacteria	Chroococcales	YP_400133	
PGK	Synechococcus sp. PCC 7002	Bacteria	Cyanobacteria	Chroococcales	YP_001734832	
PGK	Synechococcus sp. PCC 7335	Bacteria	Cyanobacteria	Chroococcales	ZP_05037854	
PGK	Prochlorococcus marinus str. MIT 9303	Bacteria	Cyanobacteria	Prochlorales	YP_001018792	
PGK	Bacillus tusciae DSM 2912	Bacteria	Firmicutes	Bacillales	YP_003589964	
PGK	Syntrophothermus lipocalidus DSM 12680	Bacteria	Firmicutes	Clostridia	YP_003703170	
PGK	Lentisphaera araneosa HTCC2155	Bacteria	Lentisphaerae	Lentisphaerales	ZP_01876284	
PGK	Acetobacter tropicalis NBRC 101654	Bacteria	Proteobacteria	Alphaproteobacteria	ZP_08644248	
PGK	Pelobacter carbinolicus DSM 2380	Bacteria	Proteobacteria	Deltaproteobacteria	YP_356751	
PGK	Magnetococcus sp. MC1	Bacteria	Proteobacteria	Magnetococcus	YP_865800	
PGAM	Neospora caninum Liverpool	Eukaryota	Alveolata	Apicomplexa	CBZ50141	
PGAM	Plasmodium falciparum 3D7	Eukaryota	Alveolata	Apicomplexa	XP_001347879	
PGAM	Paramecium tetraurelia strain d42	Eukaryota	Alveolata	Ciliophora	XP_001460229	
PGAM	Karenia brevis	Eukaryota	Alveolata	Dinophyceae	ABF73003	
PGAM	Dictyostelium discoideum AX4	Eukaryota	Amoebozoa	Mycetozoa	XP_638289	
PGAM	Guillardia theta	Eukaryota	Cryptophyta	Pyrenomonadales	Guith1_63186	retrieved from [1]
PGAM	Guillardia theta	Eukaryota	Cryptophyta	Pyrenomonadales	Guith1_82781	retrieved from [1]
PGAM	Emiliania huxleyi	Eukaryota	Haptophyceae	Isochrysidales	Emihu1_428965	retrieved from [3]
PGAM	Emiliania huxleyi	Eukaryota	Haptophyceae	Isochrysidales	Emihu1_465579	retrieved from [3]
PGAM	Naegleria gruberi strain NEGM	Eukaryota	Heterolobosea	Schizopyrenida	XP_002682122	
PGAM	Bombyx mori	Eukaryota	Metazoa	Arthropoda	NP_001037540	
PGAM	Cherax quadricarinatus	Eukaryota	Metazoa	Arthropoda	AEL23008	
PGAM	Ciona intestinalis	Eukaryota	Metazoa	Chordata	XP_002126211	
PGAM	Oikopleura dioica	Eukaryota	Metazoa	Chordata	CBY11621	
PGAM	Oreochromis niloticus	Eukaryota	Metazoa	Chordata	XP_003444047	
PGAM	Xenopus (Silurana) tropicalis	Eukaryota	Metazoa	Chordata	XP_002944910	
PGAM	Hydra magnipapillata	Eukaryota	Metazoa	Cnidaria	XP_002160107	
PGAM	Brachionus plicatilis	Eukaryota	Metazoa	Rotifera	BAI43376	
PGAM	Trichomonas vaginalis G3	Eukaryota	Parabasalia	Trichomonadida	XP_001325315	
PGAM	Bigelowiella natans	Eukaryota	Rhizaria	Cercozoa	Bigna1__36120	retrieved from [4]
PGAM	Bigelowiella natans	Eukaryota	Rhizaria	Cercozoa	Bigna1_54979	retrieved from [4]
PGAM	Bigelowiella natans	Eukaryota	Rhizaria	Cercozoa	Bigna1_75669	retrieved from [4]
PGAM	Bigelowiella natans	Eukaryota	Rhizaria	Cercozoa	Bigna1_85347	retrieved from [4]
PGAM	Bigelowiella natans	Eukaryota	Rhizaria	Cercozoa	Bigna1_88656	retrieved from [4]
PGAM	Bigelowiella natans	Eukaryota	Rhizaria	Cercozoa	Bigna1_92653	retrieved from [4]
PGAM	Bigelowiella natans	Eukaryota	Rhizaria	Cercozoa	Bigna1_92788	retrieved from [4]
PGAM	Bigelowiella natans	Eukaryota	Rhizaria	Cercozoa	Bigna1_131737	retrieved from [4]
PGAM	Bigelowiella natans	Eukaryota	Rhizaria	Cercozoa	Bigna1_134582	retrieved from [4]
PGAM	Gymnochlora stellata	Eukaryota	Rhizaria	Cercozoa	ACF24576	
PGAM	Phaeodactylum tricornutum	Eukaryota	Stramenopile	Bacillariophyta	XP_002185492.1	
PGAM	Phaeodactylum tricornutum	Eukaryota	Stramenopile	Bacillariophyta	XP_002178519.1	
PGAM	Phaeodactylum tricornutum	Eukaryota	Stramenopile	Bacillariophyta	XP_002178324.1	
PGAM	Phaeodactylum tricornutum	Eukaryota	Stramenopile	Bacillariophyta	XP_002177520.1	

PGAM	Phaeodactylum tricornutum	Eukaryota	Stramenopile	Bacillariophyta	XP_002176766.1	
PGAM	Phaeodactylum tricornutum	Eukaryota	Stramenopile	Bacillariophyta	XP_002186277.1	
PGAM	Blastocystis hominis	Eukaryota	Stramenopile	Blastocystae	CBK21549.2	
PGAM	Phytophthora infestans	Eukaryota	Stramenopile	Oomycetes	XP_002904430.1	
PGAM	Phytophthora infestans	Eukaryota	Stramenopile	Oomycetes	XP_002899444.1	
PGAM	Aureococcus anophagefferens	Eukaryota	Stramenopile	Pelagophyceae	EGB03219.1	
PGAM	Aureococcus anophagefferens	Eukaryota	Stramenopile	Pelagophyceae	EGB03220.1	
PGAM	Aureococcus anophagefferens	Eukaryota	Stramenopile	Pelagophyceae	EGB13012.1	
PGAM	Aureococcus anophagefferens	Eukaryota	Stramenopile	Pelagophyceae	EGB06466.1	
PGAM	Aureococcus anophagefferens	Eukaryota	Stramenopile	Pelagophyceae	EGB12819.1	
PGAM	Aureococcus anophagefferens	Eukaryota	Stramenopile	Pelagophyceae	EGB05650.1	
PGAM	Aureococcus anophagefferens	Eukaryota	Stramenopile	Pelagophyceae	EGB04852.1	
PGAM	Aureococcus anophagefferens	Eukaryota	Stramenopile	Pelagophyceae	EGB05651.1	
PGAM	Aureococcus anophagefferens	Eukaryota	Stramenopile	Pelagophyceae	EGB02652.1	
PGAM	Aureococcus anophagefferens	Eukaryota	Stramenopile	Pelagophyceae	EGB10687.1	
PGAM	Aureococcus anophagefferens	Eukaryota	Stramenopile	Pelagophyceae	EGB04853.1	
PGAM	Ectocarpus siliculosus	Eukaryota	Stramenopile	PX clade	CBN79496.1	
PGAM	Ectocarpus siliculosus	Eukaryota	Stramenopile	PX clade	CBN78717.1	
PGAM	Ectocarpus siliculosus	Eukaryota	Stramenopile	PX clade	CBN76135.1	
PGAM	Ectocarpus siliculosus	Eukaryota	Stramenopile	PX clade	CBN79553.1	
PGAM	Ectocarpus siliculosus	Eukaryota	Stramenopile	PX clade	CBJ28845.1	
PGAM	Ectocarpus siliculosus	Eukaryota	Stramenopile	PX clade	CBN79554.1	
PGAM	Physcomitrella patens subsp. patens	Eukaryota	Viridiplantae	Streptophyta	XP_001761673	
PGAM	Ricinus communis	Eukaryota	Viridiplantae	Streptophyta	XP_002533602	
PGAM	Sorghum bicolor	Eukaryota	Viridiplantae	Streptophyta	XP_002454871	
PGAM	Leifsonia xyli subsp. xyli str. CTCB07	Bacteria	Actinobacteria	Actinobacteridae	YP_062678	
PGAM	Saccharomonospora viridis DSM 43017	Bacteria	Actinobacteria	Actinobacteridae	YP_003133202	
PGAM	Thermovibrio ammonificans HB1	Bacteria	Aquificae	Aquificales	YP_004151946	
PGAM	Capnocytophaga sp. oral taxon 329 str. F0087	Bacteria	Bacteroidetes	Flavobacteriia	ZP_08446365	
PGAM	Roseiflexus sp. RS1	Bacteria	Chloroflexi	Chloroflexales	YP_001276039	
PGAM	Cyanothece sp. ATCC 51142	Bacteria	Cyanobacteria	Chroococcales	YP_001805578	
PGAM	Synechocystis sp. PCC 6803	Bacteria	Cyanobacteria	Chroococcales	NP_439971	
PGAM	Gloeobacter violaceus PCC 7421	Bacteria	Cyanobacteria	Gloeobacteria	NP_923717	
PGAM	Nostoc sp. PCC 7120	Bacteria	Cyanobacteria	Nostocales	NP_485150	
PGAM	Nostoc sp. PCC 7120	Bacteria	Cyanobacteria	Nostocales	NP_487378	
PGAM	Planktothrix rubescens NIVACYA 98	Bacteria	Cyanobacteria	Oscillatoriales	CAQ48287	
PGAM	Bacillus cereus R309803	Bacteria	Firmicutes	Bacillales	ZP_04289064	
PGAM	Thermoanaerobacter italicus Ab9	Bacteria	Firmicutes	Clostridia	YP_003477933	
PGAM	Lactobacillus jensenii SJ7AUS	Bacteria	Firmicutes	Lactobacillales	ZP_05865746	
PGAM	Burkholderia sp. H160	Bacteria	Proteobacteria	Betaproteobacteria	ZP_03266356	
PGAM	Collimonas fungivorans Ter331	Bacteria	Proteobacteria	Betaproteobacteria	YP_004754709	
PGAM	Leptothrix cholodnii SP6	Bacteria	Proteobacteria	Betaproteobacteria	YP_001792533	
PGAM	Methyloversatilis universalis FAM5	Bacteria	Proteobacteria	Betaproteobacteria	ZP_08505856	
PGAM	Nitrosospira multiformis ATCC 25196	Bacteria	Proteobacteria	Betaproteobacteria	YP_411816	
PGAM	Taylorella equigenitalis MCE9	Bacteria	Proteobacteria	Betaproteobacteria	YP_004129825	
PGAM	Desulfobacca acetoxidans DSM 11109	Bacteria	Proteobacteria	Deltaproteobacteria	YP_004371072	
PGAM	Pantoea ananatis LMG 20103	Bacteria	Proteobacteria	Gammaproteobacteria	YP_003519495	
PGAM	Psychromonas sp. CNPT3	Bacteria	Proteobacteria	Gammaproteobacteria	ZP_01217440	
PGAM	Sodalis glossinidius str. 'morsitans'	Bacteria	Proteobacteria	Gammaproteobacteria	YP_454574	
PGAM	Xylella fastidiosa Temecula1	Bacteria	Proteobacteria	Gammaproteobacteria	NP_779121	
PGAM	Borrelia afzelii PKo	Bacteria	Spirochaetes	Spirochaetales	YP_710109	
PGAM	Spirochaeta smaragdinae DSM 11293	Bacteria	Spirochaetes	Spirochaetales	YP_003805338	
PGAM	Anaerobaculum hydrogeniformans ATCC BAA1850	Bacteria	Synergistetes	Synergistia	ZP_06440824	
PGAM	Chthoniobacter flavus Ellin428	Bacteria	Verrucomicrobia	Spartobacteria	ZP_03129247	
PGAM	Methanosphaerula palustris E19c	Archaea	Euryarchaeota	Methanomicrobia	YP_002465729	
PGAM	Methanospirillum hungatei JF1	Archaea	Euryarchaeota	Methanomicrobia	YP_503748	
Enolase	Plasmodium knowlesi strain H	Eukaryota	Alveolata	Apicomplexa	XP_002258802	
Enolase	Toxoplasma gondii ME49	Eukaryota	Alveolata	Apicomplexa	XP_002365578	
Enolase	Paramecium tetraurelia strain d42	Eukaryota	Alveolata	Ciliophora	XP_001452406	
Enolase	Tetrahymena thermophila	Eukaryota	Alveolata	Ciliophora	XP_001014643	
Enolase	Heterocapsa triquetra	Eukaryota	Alveolata	Dinophyceae	AAU20794	
Enolase	Heterocapsa triquetra	Eukaryota	Alveolata	Dinophyceae	BAE07174	
Enolase	Heterocapsa triquetra	Eukaryota	Alveolata	Dinophyceae	AAR97555	
Enolase	Karenia brevis	Eukaryota	Alveolata	Dinophyceae	BAE07164	
Enolase	Perkinsus marinus ATCC 50983	Eukaryota	Alveolata	Perkinsea	XP_002771110	
Enolase	Entamoeba dispar SAW760	Eukaryota	Amoebozoa	Archamoebae	XP_001735821	
Enolase	Mastigamoeba balamuthi	Eukaryota	Amoebozoa	Archamoebae	Q9U615	
Enolase	Dictyostelium discoideum AX4	Eukaryota	Amoebozoa	Mycetozoa	XP_639231	
Enolase	Dictyostelium discoideum AX4	Eukaryota	Amoebozoa	Mycetozoa	XP_647650	
Enolase	Polysphondylium pallidum PN500	Eukaryota	Amoebozoa	Mycetozoa	EFA79459	
Enolase	Guillardia theta	Eukaryota	Cryptophyta	Pyrenomonadales	Guith1_82684	retrieved from [1]
Enolase	Leishmania mexicana MHOM/GT/2001/U1103	Eukaryota	Euglenozoa	Kinetoplastida	CBZ24980	
Enolase	Trypanosoma cruzi strain CL Brener	Eukaryota	Euglenozoa	Kinetoplastida	XP_819700	
Enolase	Batrachochytrium dendrobatidis JAM81	Eukaryota	Fungi	Chytridiomycota	EGF81098	
Enolase	Debaryomyces hansenii CBS767	Eukaryota	Fungi	Dikarya	XP_458559	
Enolase	Lodderomyces elongisporus NRRL YB4239	Eukaryota	Fungi	Dikarya	XP_001528121	
Enolase	Ustilago maydis 521	Eukaryota	Fungi	Dikarya	XP_759503	

Enolase	Neocallimastix frontalis	Eukaryota	Fungi	Neocallimastigomycota	P42894	
Enolase	Emiliania huxleyi	Eukaryota	Haptophyceae	Isochrysidales	Emihu1_465926	retrieved from [3]
Enolase	Emiliania huxleyi	Eukaryota	Haptophyceae	Isochrysidales	Emihu1_420746	retrieved from [3]
Enolase	Emiliania huxleyi	Eukaryota	Haptophyceae	Isochrysidales	Emihu1_444382	retrieved from [3]
Enolase	Emiliania huxleyi	Eukaryota	Haptophyceae	Isochrysidales	Emihu1_445376	retrieved from [3]
Enolase	Capsaspora owczarzaki ATCC 30864	Eukaryota	Ichthyosporea	Capsaspora	EFW47554	
Enolase	Ictalurus punctatus	Eukaryota	Metazoa	Chordata	NP_001187631	
Enolase	Hydra magnipapillata	Eukaryota	Metazoa	Cnidaria	XP_002164467	
Enolase	Doryteuthis pealeii	Eukaryota	Metazoa	Mollusca	O02654	
Enolase	Brachionus plicatilis	Eukaryota	Metazoa	Rotifera	BAI43375	
Enolase	Monocercomonoides sp. PA203	Eukaryota	Oxymonadida	Polymastigidae	ABG56067	
Enolase	Bigelowiella natans	Eukaryota	Rhizaria	Cercozoa	Bigna1_52038	retrieved from [4]
Enolase	Bigelowiella natans	Eukaryota	Rhizaria	Cercozoa	Bigna1_91743	retrieved from [4]
Enolase	Cyanidioschyzon merolae	Eukaryota	Rhodophyta	Bangiophyceae	CMK131C	retrieved from [5]
Enolase	Phaeodactylum tricornutum	Eukaryota	Stramenopile	Bacillariophyta	XP_002176181.1	
Enolase	Phaeodactylum tricornutum	Eukaryota	Stramenopile	Bacillariophyta	XP_002176196.1	
Enolase	Phaeodactylum tricornutum	Eukaryota	Stramenopile	Bacillariophyta	XP_002185511.1	
Enolase	Blastocystis hominis	Eukaryota	Stramenopile	Blastocystae	CBK20528.2	
Enolase	Blastocystis hominis	Eukaryota	Stramenopile	Blastocystae	CBK20125.2	
Enolase	Phytophthora infestans	Eukaryota	Stramenopile	Oomycetes	XP_002899300.1	
Enolase	Phytophthora infestans	Eukaryota	Stramenopile	Oomycetes	XP_002906751.1	
Enolase	Phytophthora infestans	Eukaryota	Stramenopile	Oomycetes	XP_002906750.1	
Enolase	Aureococcus anophagefferens	Eukaryota	Stramenopile	Pelagophyceae	EGB04440.1	
Enolase	Aureococcus anophagefferens	Eukaryota	Stramenopile	Pelagophyceae	EGB09778.1	
Enolase	Aureococcus anophagefferens	Eukaryota	Stramenopile	Pelagophyceae	EGB13011.1	
Enolase	Ectocarpus siliculosus	Eukaryota	Stramenopile	PX clade	CBJ32586.1	
Enolase	Ectocarpus siliculosus	Eukaryota	Stramenopile	PX clade	CBN78148.1	
Enolase	Chlamydomonas reinhardtii	Eukaryota	Viridiplantae	Chlorophyta	XP_001702971	
Enolase	Ostreococcus lucimarinus CCE9901	Eukaryota	Viridiplantae	Chlorophyta	XP_001415437	
Enolase	Arabidopsis lyrata subsp. lyrata	Eukaryota	Viridiplantae	Streptophyta	XP_002888960	
Enolase	Physcomitrella patens subsp. patens	Eukaryota	Viridiplantae	Streptophyta	XP_001751337	
Enolase	Physcomitrella patens subsp. patens	Eukaryota	Viridiplantae	Streptophyta	XP_001759508	
Enolase	Physcomitrella patens subsp. patens	Eukaryota	Viridiplantae	Streptophyta	XP_001765483	
Enolase	Spinacia oleracea	Eukaryota	Viridiplantae	Streptophyta	CAB96173	
Enolase	Corynebacterium lipophiloflavum DSM 44291	Bacteria	Actinobacteria	Actinobacteridae	ZP_03978860	
Enolase	Ktedonobacter racemifer DSM 44963	Bacteria	Chloroflexi	Ktedonobacteria	ZP_06970116	
Enolase	Crocosphaera watsonii WH 8501	Bacteria	Cyanobacteria	Chroococcales	ZP_00518338	
Enolase	Gloeobacter violaceus PCC 7421	Bacteria	Cyanobacteria	Gloeobacteria	NP_925067	
Enolase	Arthrospira platensis str. Paraca	Bacteria	Cyanobacteria	Oscillatoriales	ZP_06383786	
Enolase	Lyngbya sp. PCC 8106	Bacteria	Cyanobacteria	Oscillatoriales	ZP_01621776	
Enolase	Thermaerobacter marianensis DSM 12885	Bacteria	Firmicutes	Clostridia	YP_004103044	
Enolase	Isosphaera pallida ATCC 43644	Bacteria	Planctomycetes	Planctomycetacia	YP_004178855	
Enolase	Spirochaeta coccoides DSM 17374	Bacteria	Spirochaetes	Spirochaetales	YP_004411782	
Enolase	Thermanaerovibrio acidaminovorans DSM 6589	Bacteria	Synergistetes	Synergistia	YP_003316840	
PK	Cryptosporidium muris RN66	Eukaryota	Alveolata	Apicomplexa	XP_002142156	
PK	Plasmodium falciparum 3D7	Eukaryota	Alveolata	Apicomplexa	XP_966251	
PK	Theileria parva strain Muguga	Eukaryota	Alveolata	Apicomplexa	XP_764242	
PK	Toxoplasma gondii	Eukaryota	Alveolata	Apicomplexa	BAG14336.1	
PK	Toxoplasma gondii	Eukaryota	Alveolata	Apicomplexa	3EOE_A	
PK	Paramecium tetraurelia strain d4-2	Eukaryota	Alveolata	Ciliophora	XP_001444997	
PK	Perkinsus marinus ATCC 50983	Eukaryota	Alveolata	Perkinsea	XP_002788069	
PK	Dictyostelium fasciculatum	Eukaryota	Amoebozoa	Mycetozoa	EGG21715	
PK	Monosiga brevicollis MX1	Eukaryota	Choanoflagellida	Codonosigidae	XP_001743459	
PK	Guillardia theta	Eukaryota	Cryptophyta	Pyrenomonadales	Guith1_101689	retrieved from [1]
PK	Guillardia theta	Eukaryota	Cryptophyta	Pyrenomonadales	Guith1_98395	retrieved from [1]
PK	Guillardia theta	Eukaryota	Cryptophyta	Pyrenomonadales	Guith1_100726	retrieved from [1]
PK	Guillardia theta	Eukaryota	Cryptophyta	Pyrenomonadales	Guith1_95279	retrieved from [1]
PK	Leishmania mexicana	Eukaryota	Euglenozoa	Kinetoplastida	3E0V_A	
PK	Trypanosoma cruzi strain CL Brener	Eukaryota	Euglenozoa	Kinetoplastida	XP_820627	
PK	Schizosaccharomyces japonicus yFS275	Eukaryota	Fungi	Dikarya	XP_002173987	
PK	Emiliania huxleyi	Eukaryota	Haptophyceae	Isochrysidales	Emihu1_70323	retrieved from [3]
PK	Emiliania huxleyi	Eukaryota	Haptophyceae	Isochrysidales	Emihu1_433474	retrieved from [3]
PK	Capsaspora owczarzaki ATCC 30864	Eukaryota	Ichthyosporea	Capsaspora	EFW44693	
PK	Hydra magnipapillata	Eukaryota	Metazoa	Cnidaria	XP_002165702	
PK	Nematostella vectensis	Eukaryota	Metazoa	Cnidaria	XP_001642026	
PK	Bigelowiella natans	Eukaryota	Rhizaria	Cercozoa	Bigna1_43086	retrieved from [4]
PK	Bigelowiella natans	Eukaryota	Rhizaria	Cercozoa	Bigna1_53632	retrieved from [4]
PK	Bigelowiella natans	Eukaryota	Rhizaria	Cercozoa	Bigna1_69287	retrieved from [4]
PK	Bigelowiella natans	Eukaryota	Rhizaria	Cercozoa	Bigna1_90946	retrieved from [4]
PK	Bigelowiella natans	Eukaryota	Rhizaria	Cercozoa	Bigna1_132449	retrieved from [4]
PK	Bigelowiella natans	Eukaryota	Rhizaria	Cercozoa	Bigna1_139168	retrieved from [4]
PK	Bigelowiella natans	Eukaryota	Rhizaria	Cercozoa	Bigna1_141401	retrieved from [4]
PK	Bigelowiella natans	Eukaryota	Rhizaria	Cercozoa	Bigna1_142509	retrieved from [4]
PK	Cyanidioschyzon merolae	Eukaryota	Rhodophyta	Bangiophyceae	CMP260C	retrieved from [5]
PK	Cyanidioschyzon merolae	Eukaryota	Rhodophyta	Bangiophyceae	CMK041C	retrieved from [5]
PK	Cyanidioschyzon merolae	Eukaryota	Rhodophyta	Bangiophyceae	CMC021C	retrieved from [5]
PK	Cyanidioschyzon merolae	Eukaryota	Rhodophyta	Bangiophyceae	CMA030C	retrieved from [5]

PK	Porphyridium cruentum	Eukaryota	Rhodophyta	Bangiophyceae	Porcr1_lf_Contig823_2	retrieved from [6]
PK	Phaeodactylum tricornutum	Eukaryota	Stramenopile	Bacillariophyta	XP_002183584.1	
PK	Phaeodactylum tricornutum	Eukaryota	Stramenopile	Bacillariophyta	XP_002184341.1	
PK	Phaeodactylum tricornutum	Eukaryota	Stramenopile	Bacillariophyta	XP_002183637.1	
PK	Phaeodactylum tricornutum	Eukaryota	Stramenopile	Bacillariophyta	XP_002180140.1	
PK	Phaeodactylum tricornutum	Eukaryota	Stramenopile	Bacillariophyta	XP_002180143.1	
PK	Phaeodactylum tricornutum	Eukaryota	Stramenopile	Bacillariophyta	XP_002183769.1	
PK	Phaeodactylum tricornutum	Eukaryota	Stramenopile	Bacillariophyta	XP_002180144.1	
PK	Phaeodactylum tricornutum	Eukaryota	Stramenopile	Bacillariophyta	XP_002182818.1	
PK	Blastocystis hominis	Eukaryota	Stramenopile	Blastocystae	CBK20192.2	
PK	Phytophthora infestans	Eukaryota	Stramenopile	Oomycetes	XP_002904433.1	
PK	Phytophthora infestans	Eukaryota	Stramenopile	Oomycetes	XP_002903650.1	
PK	Phytophthora infestans	Eukaryota	Stramenopile	Oomycetes	XP_002903652.1	
PK	Phytophthora infestans	Eukaryota	Stramenopile	Oomycetes	XP_002907650.1	
PK	Phytophthora infestans	Eukaryota	Stramenopile	Oomycetes	XP_002903651.1	
PK	Aureococcus anophagefferens	Eukaryota	Stramenopile	Pelagophyceae	EGB05272.1	
PK	Aureococcus anophagefferens	Eukaryota	Stramenopile	Pelagophyceae	EGB08740.1	
PK	Aureococcus anophagefferens	Eukaryota	Stramenopile	Pelagophyceae	EGB09412.1	
PK	Aureococcus anophagefferens	Eukaryota	Stramenopile	Pelagophyceae	EGB05557.1	
PK	Aureococcus anophagefferens	Eukaryota	Stramenopile	Pelagophyceae	EGB06755.1	
PK	Aureococcus anophagefferens	Eukaryota	Stramenopile	Pelagophyceae	EGB09670.1	
PK	Ectocarpus siliculosus	Eukaryota	Stramenopile	PX clade	CBJ30464.1	
PK	Ectocarpus siliculosus	Eukaryota	Stramenopile	PX clade	CBJ32589.1	
PK	Ectocarpus siliculosus	Eukaryota	Stramenopile	PX clade	CBN80295.1	
PK	Chlamydomonas reinhardtii	Eukaryota	Viridiplantae	Chlorophyta	XP_001693008	
PK	Chlamydomonas reinhardtii	Eukaryota	Viridiplantae	Chlorophyta	XP_001700637	
PK	Micromonas sp. RCC299	Eukaryota	Viridiplantae	Chlorophyta	XP_002500945	
PK	Micromonas sp. RCC299	Eukaryota	Viridiplantae	Chlorophyta	XP_002503288	
PK	Micromonas sp. RCC299	Eukaryota	Viridiplantae	Chlorophyta	XP_002503101	
PK	Physcomitrella patens subsp. patens	Eukaryota	Viridiplantae	Streptophyta	XP_001775912	
PK	Selaginella moellendorffii	Eukaryota	Viridiplantae	Streptophyta	XP_002980394	
PK	Acaryochloris marina MBIC11017	Bacteria	Cyanobacteria	Chroococcales	YP_001515306	
PK	Cyanothece sp. PCC 8801	Bacteria	Cyanobacteria	Chroococcales	YP_002372744	
PK	Microcystis aeruginosa NIES-843	Bacteria	Cyanobacteria	Chroococcales	YP_001660558	
PK	Synechococcus elongatus PCC 6301	Bacteria	Cyanobacteria	Chroococcales	YP_172116	
PK	Synechococcus sp. RCC307	Bacteria	Cyanobacteria	Chroococcales	YP_001227655	
PK	Synechococcus sp. RS9917	Bacteria	Cyanobacteria	Chroococcales	ZP_01080264	
PK	Gloeobacter violaceus PCC 7421	Bacteria	Cyanobacteria	Gloeobacteria	NP_926441	
PK	Anaerofustis stercorihominis DSM 17244	Bacteria	Firmicutes	Clostridia	ZP_02862770	
PK	Turicibacter sanguinis PC909	Bacteria	Firmicutes	Erysipelotrichi	ZP_06622396	
PK	Ilyobacter polytropus DSM 2926	Bacteria	Fusobacteria	Fusobacteriales	YP_003967878	
PK	alpha proteobacterium BAL199	Bacteria	Proteobacteria	Alphaproteobacteria	ZP_02189178	
PK	Candidatus Puniceispirillum marinum IMCC1322	Bacteria	Proteobacteria	Alphaproteobacteria	YP_003551764	
PK	Aeromonas salmonicida subsp. salmonicida A449	Bacteria	Proteobacteria	Gammaproteobacteria	YP_001142172	
PK	Photorhabdus luminescens subsp. laumondii TTO1	Bacteria	Proteobacteria	Gammaproteobacteria	NP_929848	
PK	Psychromonas sp. CNPT3	Bacteria	Proteobacteria	Gammaproteobacteria	ZP_01215694	
PK	Magnetococcus sp. MC-1	Bacteria	Proteobacteria	Magnetococcus	YP_864655	
PK	Synergistetes bacterium SGP1	Bacteria	Synergistetes	SGP1	CBL28857	
PK	Thermosipho africanus TCF52B	Bacteria	Thermotogae	Thermotogales	YP_002334992	

[1]: http://genome.jgi-psf.org/Guith1/Guith1.home.html

[2]: http://amoebidia.bcm.umontreal.ca/pepdb/searches/organism.php?orgID=EL

[3]: http://genome.jgi-psf.org/Emihu1/Emihu1.home.html

[4]: http://genome.jgi-psf.org/Bigna1/Bigna1.home.html

[5]: http://merolae.biol.s.u-tokyo.ac.jp

[6]: Chan CX, Yang EC, Banerjee T, Yoon HS, Martone PT, Estevez JM and Bhattacharya (2011) Current Biology, 21(4): 328-333
