# Supplementary material for: Broad Distribution of TPI-GAPDH Fusion Proteins among Eukaryotes: Evidence for Glycolytic Reactions in the Mitochondrion?
Source: PLoS One. 2012 Dec 20;7(12):e52340. doi: 10.1371/journal.pone.0052340 (PMC3527533; doi:10.1371/journal.pone.0052340)
Supplement: Table S2 — TPI-GAPDH subcellular localization predictions. (PDF) [file pone.0052340.s006.pdf]

Table S2.TPI-GAPDH subcellular localization predictions

Organism	GenBank accession number / reference	TargetP (Reliability class)	iPSORT	Predotar	PredSL
Paulinella chromatophora M0880/a	JQ783118-JQ783119	M (2)	Mitochondrial Transit Peptide	mitochondrial	mitochondrion
Phaeodactylum tricornutum	AAF34330	M (1)	Mitochondrial Transit Peptide	possibly mitochondrial	mitochondrion
Odontella sinensis	AAF34328	M (2)	Mitochondrial Transit Peptide	mitochondrial	mitochondrion
Phytophthora infestans	Liaud et al. 2000* (Original: X64537)	M (3)	Mitochondrial Transit Peptide	possibly mitochondrial	mitochondrion
Blastocystis hominis	CBK20353	M (3)	Mitochondrial Transit Peptide	mitochondrial	mitochondrion
Ectocarpus siliculosus (revised)**	See main text	M (2)	Mitochondrial Transit Peptide	possibly mitochondrial	mitochondrion
Thecamonas trahens	ADVD01000324 (WGS sequence contig)	M (2)	Mitochondrial Transit Peptide	mitochondrial	mitochondrion
Achlya bisexualis	AAF44720	M (3)	Mitochondrial Transit Peptide	mitochondrial	mitochondrion
Hyaloperonospora arabidopsidis	ABWE01000065 (WGS sequence contig)	M (2)	Mitochondrial Transit Peptide	mitochondrial	mitochondrion
Saprolegnia parasitica	ADCG01002396 (WGS sequence contig)	M (1)	Mitochondrial Transit Peptide	possibly mitochondrial	mitochondrion
Pythium ultimum	ADOS01000576 (WGS sequence contig)	M (3)	Mitochondrial Transit Peptide	mitochondrial	mitochondrion

* Liaud, M. F., C. Lichtle, K. Apt, W. Martin, and R. Cerff. 2000. Compartment-specific isoforms of TPI and GAPDH are imported into diatom mitochondria as a fusion protein: evidence in favor of a mitochondrial origin of the eukaryotic glycolytic pathway. Mol Biol Evol 17:213-223.

** Alternative protein model postulated in this study, See main text for details.
