# Supplementary material for: Broad Distribution of TPI-GAPDH Fusion Proteins among Eukaryotes: Evidence for Glycolytic Reactions in the Mitochondrion?
Source: PLoS One. 2012 Dec 20;7(12):e52340. doi: 10.1371/journal.pone.0052340 (PMC3527533; doi:10.1371/journal.pone.0052340)
Supplement: Table S3 — Subcellular localization predictions for various glycolytic enzymes. (PDF) [file pone.0052340.s007.pdf]

Table S3. Subcellular localization predictions for various glycolytic enzymes

Organism	Enzyme	GenBank accession number / JGI protein ID	'plant' setting				non-plant' setting				Cumulative prediction
			TargetP (Reliability class)	iPSORT	Predotar	PredSL	TargetP (Reliability class)	iPSORT	Predotar	PredSL	
Bigelowiella natans	Enolase	52038	M (5)	Other	none	other	_ (4)	Other	none	other	
Bigelowiella natans	Enolase	91743	_ (3)	Other	none	chloroplast	_ (2)	Other	none	other	
Phaeodactylum tricornutum	Enolase	XP_002176181.1	M (3)	Mitochondrial Transit Peptide	possibly mitochondrial	mitochondrion	M (1)	Mitochondrial Transit Peptide	possibly mitochondrial	mitochondrion	mitochondrion
Phaeodactylum tricornutum	Enolase	XP_002176196.1	M (3)	Mitochondrial Transit Peptide	possibly mitochondrial	mitochondrion	M (1)	Mitochondrial Transit Peptide	possibly mitochondrial	mitochondrion	mitochondrion
Aureococcus anophagefferens	Enolase	EGB04440.1	M (2)	Mitochondrial Transit Peptide	none	mitochondrion	M (2)	Mitochondrial Transit Peptide	none	mitochondrion	
Phaeodactylum tricornutum	Enolase	XP_002185511.1	C (3)	Signal Peptide	ER	chloroplast	S (3)	Signal Peptide	ER	secreted	
Ectocarpus siliculosus	Enolase	CBJ32586.1	M (3)	Mitochondrial Transit Peptide	mitochondrial	mitochondrion	M (1)	Mitochondrial Transit Peptide	mitochondrial	mitochondrion	mitochondrion
Aureococcus anophagefferens	Enolase	EGB09778.1	_ (4)	Other	none	other	_ (1)	Other	none	other	
Ectocarpus siliculosus	Enolase	CBN78148.1	S (3)	Signal Peptide	ER	secreted	S (1)	Signal Peptide	ER	secreted	
Phytophthora infestans	Enolase	XP_002899300.1	M (3)	Mitochondrial Transit Peptide	mitochondrial	mitochondrion	M (2)	Mitochondrial Transit Peptide	mitochondrial	mitochondrion	mitochondrion
Aureococcus anophagefferens	Enolase	EGB13011.1	C (4)	Chloroplast Transit Peptide	plastid	other	M (3)	Other	none	other	
Phytophthora infestans	Enolase	XP_002906751.1	_ (3)	Other	possibly mitochondrial	other	_ (4)	Other	possibly mitochondrial	other	
Phytophthora infestans	Enolase	XP_002906750.1	_ (3)	Other	possibly mitochondrial	other	_ (4)	Other	possibly mitochondrial	other	
Blastocystis hominis	Enolase	CBK20528.2	M (4)	Mitochondrial Transit Peptide	mitochondrial	mitochondrion	M (2)	Mitochondrial Transit Peptide	mitochondrial	mitochondrion	mitochondrion
Blastocystis hominis	Enolase	CBK20125.2	_ (2)	Other	none	other	_ (2)	Other	none	other	
Bigelowiella natans	PGAM	36120	M (3)	Mitochondrial Transit Peptide	none	mitochondrion	M (3)	Mitochondrial Transit Peptide	none	mitochondrion	
Bigelowiella natans	PGAM	54979	M (4)	Other	none	mitochondrion	_ (4)	Other	none	other	
Bigelowiella natans	PGAM	75669	_ (2)	Other	none	mitochondrion	_ (2)	Other	none	mitochondrion	
Bigelowiella natans	PGAM	85347	M (3)	Mitochondrial Transit Peptide	possibly mitochondrial	mitochondrion	M (3)	Mitochondrial Transit Peptide	possibly mitochondrial	mitochondrion	mitochondrion
Bigelowiella natans	PGAM	87481	_ (2)	Other	none	other	_ (1)	Other	none	other	
Bigelowiella natans	PGAM	88656	_ (5)	Mitochondrial Transit Peptide	none	other	_ (4)	Other	none	other	
Bigelowiella natans	PGAM	92653	S (5)	Signal Peptide	ER	mitochondrion	S (4)	Signal Peptide	ER	secreted	
Bigelowiella natans	PGAM	92788	M (1)	Mitochondrial Transit Peptide	mitochondrial	mitochondrion	M (1)	Mitochondrial Transit Peptide	mitochondrial	mitochondrion	mitochondrion
Bigelowiella natans	PGAM	131737	_ (3)	Other	none	secreted	_ (2)	Other	none	other	
Bigelowiella natans	PGAM	134582	_ (3)	Other	none	mitochondrion	_ (2)	Other	none	other	
Phaeodactylum tricornutum	PGAM	XP_002185492.1	M (4)	Mitochondrial Transit Peptide	ER	other	S (4)	Signal Peptide	ER	secreted	
Phytophthora infestans	PGAM	XP_002904430.1	_ (4)	Other	none	other	_ (2)	Other	none	other	
Phytophthora infestans	PGAM	XP_002899444.1	M (1)	Mitochondrial Transit Peptide	mitochondrial	mitochondrion	M (1)	Mitochondrial Transit Peptide	mitochondrial	mitochondrion	mitochondrion
Ectocarpus siliculosus	PGAM	CBN79496.1	M (1)	Mitochondrial Transit Peptide	mitochondrial	mitochondrion	M (2)	Mitochondrial Transit Peptide	mitochondrial	mitochondrion	mitochondrion
Blastocystis hominis	PGAM	CBK21549.2	M (5)	Mitochondrial Transit Peptide	none	mitochondrion	_ (5)	Other	none	mitochondrion	
Ectocarpus siliculosus	PGAM	CBN78717.1	M (5)	Signal Peptide	ER	secreted	S (2)	Signal Peptide	ER	secreted	
Phaeodactylum tricornutum	PGAM	XP_002178519.1	M (4)	Mitochondrial Transit Peptide	mitochondrial	mitochondrion	M (1)	Mitochondrial Transit Peptide	mitochondrial	mitochondrion	mitochondrion
Aureococcus anophagefferens	PGAM	EGB03219.1	M (2)	Mitochondrial Transit Peptide	mitochondrial	mitochondrion	M (2)	Mitochondrial Transit Peptide	mitochondrial	mitochondrion	mitochondrion
Aureococcus anophagefferens	PGAM	EGB03220.1	_ (3)	Other	-	mitochondrion	_ (2)	Signal Peptide	-	other	
Aureococcus anophagefferens	PGAM	EGB13012.1	_ (2)	Other	-	mitochondrion	_ (2)	Other	-	other	
Aureococcus anophagefferens	PGAM	EGB06466.1	_ (3)	Other	none	mitochondrion	_ (2)	Other	none	other	
Ectocarpus siliculosus	PGAM	CBN76135.1	_ (2)	Other	none	other	_ (2)	Other	none	other	
Phaeodactylum tricornutum	PGAM	XP_002178324.1	_ (5)	Mitochondrial Transit Peptide	none	mitochondrion	M (3)	Other	none	other	
Aureococcus anophagefferens	PGAM	EGB12819.1	M (5)	Other	-	other	_ (4)	Other	-	other	
Phaeodactylum tricornutum	PGAM	XP_002177520.1	C (4)	Mitochondrial Transit Peptide	mitochondrial	mitochondrion	M (2)	Mitochondrial Transit Peptide	mitochondrial	mitochondrion	
Phaeodactylum tricornutum	PGAM	XP_002176766.1	S (1)	Signal Peptide	ER	secreted	S (1)	Signal Peptide	ER	secreted	
Ectocarpus siliculosus	PGAM	CBN79553.1	_ (4)	Mitochondrial Transit Peptide	none	mitochondrion	_ (3)	Other	none	other	
Ectocarpus siliculosus	PGAM	CBJ28845.1	_ (3)	Other	none	mitochondrion	_ (2)	Other	none	other	
Aureococcus anophagefferens	PGAM	EGB05650.1	_ (2)	Other	-	secreted	_ (2)	Other	-	other	
Aureococcus anophagefferens	PGAM	EGB04852.1	_ (2)	Other	-	other	_ (2)	Other	-	other	
Aureococcus anophagefferens	PGAM	EGB05651.1	_ (3)	Other	-	other	_ (2)	Other	-	other	
Aureococcus anophagefferens	PGAM	EGB02652.1	_ (2)	Other	-	other	_ (2)	Other	-	other	
Aureococcus anophagefferens	PGAM	EGB10687.1	M (2)	Mitochondrial Transit Peptide	ER	mitochondrion	M (5)	Mitochondrial Transit Peptide	ER	secreted	
Ectocarpus siliculosus	PGAM	CBN79554.1	_ (2)	Other	none	secreted	_ (3)	Other	none	secreted	
Phaeodactylum tricornutum	PGAM	XP_002186277.1	M (3)	Mitochondrial Transit Peptide	ER	secreted	S (3)	Mitochondrial Transit Peptide	ER	mitochondrion	
Aureococcus anophagefferens	PGAM	EGB04853.1	_ (3)	Other	-	mitochondrion	_ (3)	Other	-	other	
Bigelowiella natans	PGK	92275	_ (2)	Other	none	other	_ (2)	Other	none	other	
Bigelowiella natans	PGK	92730	S (1)	Signal Peptide	ER	other	S (1)	Signal Peptide	ER	secreted	
Phaeodactylum tricornutum	PGK	XP_002183701.1	M (3)	Mitochondrial Transit Peptide	mitochondrial	mitochondrion	M (1)	Mitochondrial Transit Peptide	mitochondrial	mitochondrion	mitochondrion
Phytophthora infestans	PGK	XP_002908759.1	M (2)	Mitochondrial Transit Peptide	mitochondrial	mitochondrion	M (1)	Mitochondrial Transit Peptide	mitochondrial	mitochondrion	mitochondrion
Ectocarpus siliculosus	PGK	CBN73855.1	M (3)	Mitochondrial Transit Peptide	mitochondrial	mitochondrion	M (2)	Mitochondrial Transit Peptide	mitochondrial	mitochondrion	mitochondrion
Ectocarpus siliculosus	PGK	CBN75134.1	_ (2)	Other	none	other	_ (1)	Other	none	other	
Ectocarpus siliculosus	PGK	CBN75135.1	_ (2)	Other	none	other	_ (1)	Other	none	other	
Aureococcus anophagefferens	PGK	EGB10290.1	M (4)	Mitochondrial Transit Peptide	possibly mitochondrial	mitochondrion	M (2)	Mitochondrial Transit Peptide	possibly mitochondrial	mitochondrion	mitochondrion
Phaeodactylum tricornutum	PGK	XP_002182409.1	S (2)	Signal Peptide	ER	secreted	S (3)	Signal Peptide	ER	secreted	
Ectocarpus siliculosus	PGK	CBN79775.1	S (3)	Signal Peptide	ER	mitochondrion	S (2)	Signal Peptide	ER	secreted	
Blastocystis hominis	PGK	CBK20833.2	M (2)	Mitochondrial Transit Peptide	mitochondrial	mitochondrion	M (2)	Mitochondrial Transit Peptide	mitochondrial	mitochondrion	mitochondrion
Ectocarpus siliculosus	PGK	CBN75623.1	S (4)	Signal Peptide	possibly ER	chloroplast	S (5)	Signal Peptide	possibly ER	secreted	
Aureococcus anophagefferens	PGK	EGB09450.1	_ (3)	Other	none	other	_ (3)	Other	none	other	
Phaeodactylum tricornutum	PGK	XP_002182724.1	_ (4)	Other	none	other	_ (4)	Other	none	other	
Bigelowiella natans	PK	53632	_ (3)	Other	none	other	_ (2)	Other	none	other	

Bigelowiella natans	PK	69287	_ (5)	Chloroplast Transit Peptide	none	secreted	S (4)	Other	none	secreted	
Bigelowiella natans	PK	80566	M (1)	Mitochondrial Transit Peptide	mitochondrial	mitochondrion	M (2)	Mitochondrial Transit Peptide	mitochondrial	mitochondrion	mitochondrion
Bigelowiella natans	PK	90946	_ (3)	Other	none	mitochondrion	_ (5)	Mitochondrial Transit Peptide	none	mitochondrion	
Bigelowiella natans	PK	128633	_ (4)	Other	none	other	_ (2)	Other	none	other	
Bigelowiella natans	PK	132449	C (4)	Other	possibly plastid	mitochondrion	_ (3)	Other	none	other	
Bigelowiella natans	PK	139168	_ (5)	Other	none	other	_ (2)	Other	none	other	
Bigelowiella natans	PK	141401	M (4)	Mitochondrial Transit Peptide	possibly mitochondrial	mitochondrion	M (4)	Mitochondrial Transit Peptide	possibly mitochondrial	mitochondrion	mitochondrion
Phaeodactylum tricornutum	PK	XP_002183584.1	M (1)	Mitochondrial Transit Peptide	mitochondrial	mitochondrion	M (1)	Mitochondrial Transit Peptide	mitochondrial	mitochondrion	mitochondrion
Aureococcus anophagefferens	PK	EGB05272.1	M (1)	Mitochondrial Transit Peptide	mitochondrial	mitochondrion	M (2)	Mitochondrial Transit Peptide	mitochondrial	mitochondrion	mitochondrion
Phytophthora infestans	PK	XP_002904433.1	M (1)	Mitochondrial Transit Peptide	mitochondrial	mitochondrion	M (2)	Mitochondrial Transit Peptide	mitochondrial	mitochondrion	mitochondrion
Ectocarpus siliculosus	PK	CBJ30464.1	M (4)	Mitochondrial Transit Peptide	none	mitochondrion	S (5)	Other	none	mitochondrion	
Phaeodactylum tricornutum	PK	XP_002184341.1	M (2)	Mitochondrial Transit Peptide	mitochondrial	mitochondrion	M (1)	Mitochondrial Transit Peptide	mitochondrial	mitochondrion	mitochondrion
Phaeodactylum tricornutum	PK	XP_002183637.1	_ (3)	Other	none	other	_ (2)	Other	none	other	
Phytophthora infestans	PK	XP_002903650.1	M (3)	Mitochondrial Transit Peptide	mitochondrial	mitochondrion	_ (2)	Mitochondrial Transit Peptide	mitochondrial	other	
Phytophthora infestans	PK	XP_002903652.1	_ (4)	Other	none	mitochondrion	_ (3)	Other	none	other	
Ectocarpus siliculosus	PK	CBJ32589.1	_ (2)	Other	none	secreted	_ (3)	Other	none	other	
Aureococcus anophagefferens	PK	EGB08740.1	_ (5)	Other	none	chloroplast	_ (5)	Other	none	other	
Phaeodactylum tricornutum	PK	XP_002180140.1	_ (3)	Other	none	secreted	_ (2)	Other	none	other	
Phytophthora infestans	PK	XP_002907650.1	M (4)	Mitochondrial Transit Peptide	none	mitochondrion	_ (5)	Mitochondrial Transit Peptide	none	mitochondrion	
Phaeodactylum tricornutum	PK	XP_002180143.1	_ (3)	Other	none	secreted	_ (2)	Other	none	other	
Phaeodactylum tricornutum	PK	XP_002183769.1	_ (3)	Chloroplast Transit Peptide	none	other	_ (4)	Other	none	other	
Aureococcus anophagefferens	PK	EGB09412.1	S (3)	Mitochondrial Transit Peptide	none	other	_ (5)	Other	none	other	
Phaeodactylum tricornutum	PK	XP_002180144.1	M (2)	Chloroplast Transit Peptide	possibly ER	other	_ (5)	Other	possibly ER	other	
Phytophthora infestans	PK	XP_002903651.1	M (4)	Other	none	mitochondrion	M (4)	Other	none	other	
Aureococcus anophagefferens	PK	EGB05557.1	M (2)	Mitochondrial Transit Peptide	mitochondrial	mitochondrion	M (1)	Mitochondrial Transit Peptide	mitochondrial	mitochondrion	mitochondrion
Blastocystis hominis	PK	CBK20192.2	_ (3)	Other	none	other	_ (2)	Other	none	other	
Ectocarpus siliculosus	PK	CBN80295.1	C (1)	Signal Peptide	ER	chloroplast	S (2)	Signal Peptide	ER	secreted	
Phaeodactylum tricornutum	PK	XP_002182818.1	C (3)	Signal Peptide	ER	chloroplast	M (5)	Signal Peptide	ER	secreted	
Aureococcus anophagefferens	PK	EGB06755.1	M (4)	Chloroplast Transit Peptide	ER	mitochondrion	M (4)	Signal Peptide	ER	secreted	
Aureococcus anophagefferens	PK	EGB09670.1	_ (3)	Chloroplast Transit Peptide	none	other	_ (2)	Other	none	other	
